# Deciphering bone marrow engraftment after allogeneic stem cell transplantation in humans using single-cell analyses

**DOI:** 10.1172/JCI180331

**Published:** 2024-08-29

**Authors:** Jennifer Bordenave, Dorota Gajda, David Michonneau, Nicolas Vallet, Mathieu Chevalier, Emmanuelle Clappier, Pierre Lemaire, Stéphanie Mathis, Marie Robin, Aliénor Xhaard, Flore Sicre de Fontbrune, Aurélien Corneau, Sophie Caillat-Zucman, Regis Peffault de Latour, Emmanuel Curis, Gérard Socié

**Affiliations:** 1 INSERM UMR 976, Université Paris Cité, Paris, France.; 2 UR 7537 BioSTM, Faculté de Pharmacie, Université Paris Cité, Paris, France.; 3 Assistance Publique–Hôpitaux de Paris (APHP), Hématologie Greffe, Hôpital Saint Louis, Paris, France.; 4 UFR de Médecine, Université Paris Cité, Paris, France.; 5 APHP, Laboratoire d’Hématologie, Hôpital Saint Louis, Saint-Louis, France.; 6 Plateforme de Cytométrie de la Pitié-Salpétrière (CyPS), UMS037-PASS, Paris, France.; 7 Faculté de Médecine, Sorbonne Université, Paris, France.; 8 APHP, Laboratoire d’Immunologie, Hôpital Saint Louis, Saint-Louis, France.; 9 APHP, Laboratoire d’Hématologie, Hôpital Lariboisière, Paris, France.

**Keywords:** Hematology, Immunology, Stem cell transplantation

## Abstract

**BACKGROUND:**

Donor cell engraftment is a prerequisite of successful allogeneic hematopoietic stem cell transplantation. Based on peripheral blood analyses, it is characterized by early myeloid recovery and T and B cell lymphopenia. However, cellular networks associated with bone marrow engraftment of allogeneic human cells have been poorly described.

**METHODS:**

Mass cytometry and CITE-Seq analyses were performed on bone marrow cells 3 months after transplantation in patients with acute myelogenous leukemia.

**RESULTS:**

Mass cytometric analyses in 26 patients and 20 healthy controls disclosed profound alterations in myeloid and B cell progenitors, with a shift toward terminal myeloid differentiation and decreased B cell progenitors. Unsupervised analysis separated recipients into 2 groups, one of them being driven by previous graft-versus-host disease (R2 patients). We then used single-cell CITE-Seq to decipher engraftment, which resolved 36 clusters, encompassing all bone marrow cellular components. Hematopoiesis in transplant recipients was sustained by committed myeloid and erythroid progenitors in a setting of monocyte-, NK cell–, and T cell–mediated inflammation. Gene expression revealed major pathways in transplant recipients, namely, TNF-α signaling via NF-κB and the IFN-γ response. The hallmark of allograft rejection was consistently found in clusters from transplant recipients, especially in R2 recipients.

**CONCLUSION:**

Bone marrow cell engraftment of allogeneic donor cells is characterized by a state of emergency hematopoiesis in the setting of an allogeneic response driving inflammation.

**FUNDING:**

This study was supported by the French National Cancer Institute (Institut National du Cancer; PLBIO19-239) and by an unrestricted research grant by Alexion Pharmaceuticals.

## Abstract 2

Decipher engraftment of allogeneic cells within the bone marrow

## Graphical abstract

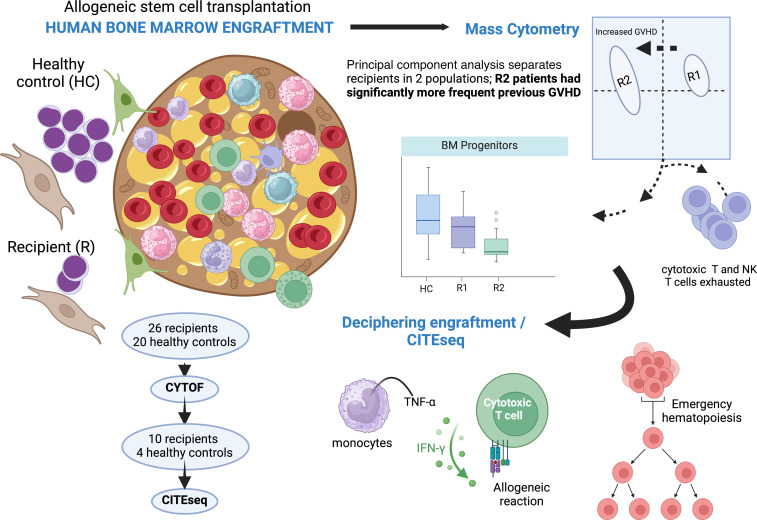

## Introduction

Since early reports of allogeneic hematopoietic stem cell (HSC) transplantation almost 60 years ago, over 750,000 procedures have been performed worldwide (1). Engraftment is a prerequisite for successful transplantation and has been defined historically by sustained increase of neutrophils, and hemoglobin and platelet levels above those requiring transfusions.

Tracking of murine HSCs in vivo showed their contribution during steady state, and single murine as well as human HSCs can successfully reconstitute a congenic or immune-deficient xenogeneic murine host after transplantation (2–6). However, studying the fate of transplanted HSCs in humans turns out to be very challenging. Knowledge about the engraftment kinetics and contribution of human HSCs during hematopoietic recovery is currently limited to young patients who underwent gene therapy, in which tracking of the retroviral integration site was used to track engraftment and clonal cell populations mostly inferred from peripheral blood mononuclear cell (PBMC) analyses (2, 7–9).

After allogeneic HSC transplantation (HSCT), in humans, engraftment has almost exclusively been studied from blood samples, showing early donor-derived monocyte and granulocyte engraftment (10). Early B cell reconstitution has been studied in detail (11–15) but again from blood samples. Finally, circulating T cells at engraftment almost exclusively reflect homeostatic proliferation of transferred mature cells since de novo thymopoiesis is delayed for months after initial engraftment (10). Bone marrow cell studies have been hampered for years by technological hurdles mostly related to limited numbers of cells at the time of engraftment. Single-cell technologies now allow in-depth study of cell populations in human immunology with relatively limited material amounts (16). Recently, peripheral blood B cell reconstitution has been extensively studied in patients with chronic graft-versus-host disease (GVHD) using single-cell transcriptomics and has highlighted how alloimmune response can impact B cell development (17). We thus took advantage of these new advances to explore bone marrow cell populations at the time of engraftment after allogeneic HSCT in humans.

We focused our research on 3-month analyses of engrafted bone marrow cells. Although initially oriented on B cell engraftment, our first results led us to consider the interplay of the other hematopoietic compartments, i.e., myeloid progenitors and T and NK cells. Using single-cell approaches to study these bone marrow cell populations, we propose that engraftment of allogeneic cells within the bone marrow is characterized by a state evoking emergency hematopoiesis within the setting of immune-associated inflammation.

## Results

### Peripheral blood assessment.

In 42 adult patients with myeloid malignancies, bone marrow cells were analyzed 3 months after HSCT and were included in this study (Supplemental Table 1; supplemental material available online with this article; https://doi.org/10.1172/JCI180331DS1). Of these 42 patients, we analyzed PBMCs in 38 patients (Supplemental Figure 1). Although reaching the criteria for engraftment, they exhibited some degrees of cytopenia, and both bone marrow and PBMC chimerism analyses disclosed that more than 95% of the cells were of donor-cell origin (Supplemental Figure 2).

B cell populations were first studied by mass cytometry in PBMCs. As already reported by us (18, 19) and others (14, 15), recipients with transplants had significantly increased percentages of transitional B cells and decreased percentages of naive and memory B cells as compared with healthy controls (Supplemental Figure 3).

### Deciphering bone marrow cell subsets using mass cytometry.

Our initial aim was to specifically study bone marrow B cell progenitors to determine whether peripheral B cell lymphopenia could be explained by defects during B lymphopoiesis. We thus designed a panel according to previously published studies (20, 21). Twenty-six patients and 12 healthy controls were included. Patient and transplant characteristics are summarized in Supplemental Table 2. Bone marrow standard cytology results are displayed in Supplemental Table 3. All patients were in complete cytological remission of their original hematological malignancy, and bone marrow chimerism analyses disclosed almost full donor chimerism (Supplemental Figure 2).

### General description of cellular populations.

**Figure 1:**
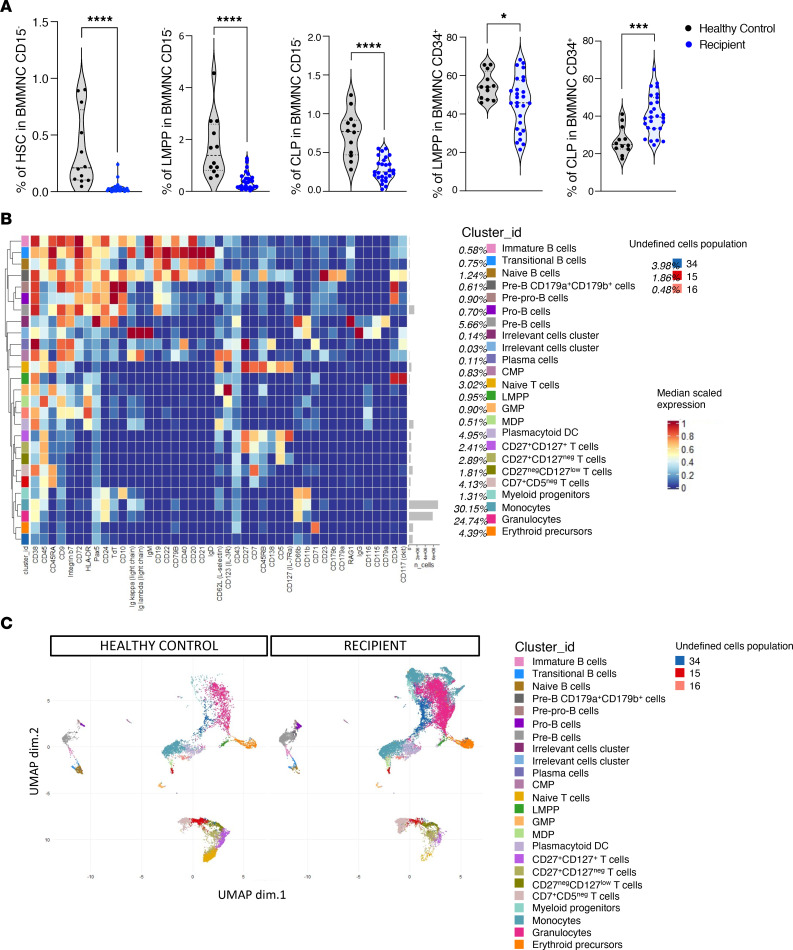
Characterization of the different populations of human cells in bone marrow. (**A**) Quantification of percentages of hematopoietic stem cells (HSCs), LMPPs, and common lymphoid progenitors (CLPs) from CD15^–^ bone marrow mononuclear cells (BMMNCs) and of LMPPs and CLPs from CD34^+^CD15^–^ BMMNCs. The gating strategy used to sort early progenitor cells from human CD15^–^ BMMNCs is summarized in Supplemental Figure 5, A and B. Twenty-six patients with acute myeloid leukemia/myelodysplastic syndrome were included in the cohort. BMMNCs were collected at 3 months after HSCT and analyzed. Twelve healthy controls were also analyzed. HSCs were defined as Lin^–^CD34^+^CD38^–^CD45RA^–^, LMPPs as CD34^+^CD38^+^CD117^+^CD127^–^, and CLPs as CD34^+^CD38^+^CD117^–^CD127^–^. The heatmap shows the median in patients (blue) compared with healthy controls (black). (**B**) Heatmap showing median expression intensities of each protein marker (columns) for each detected cluster (rows) using FlowSOM algorithm, with 24 indicated relevant metaclusters. (**C**) UMAP plot generated from an equal subsampling by subset from CD15^–^ BMMNCs from healthy controls (*n* = 12) and recipients (*n* = 26) (42 surface and 2 intracellular markers). Clusters are color-coded. For **A** and **B**, Mann-Whitney tests: *P < 0.05; ***P < 0.001; ****P < 0.0001.

The bone marrow populations were assigned as in ref. 21 and are summarized in Supplemental Figure 4 and Supplemental Table 4A (antibody panel) and Supplemental Table 4B (phenotypic definition). Since many bone marrow cells at engraftment were mature granulocytes, a gating strategy was used to exclude CD15-positive cells (Supplemental Figure 5A). Using the gating strategy described in Supplemental Figure 5B, the percentages of early progenitors that were HSCs (Lin^–^CD34^+^CD38^–^CD45RA^–^), bipotent lymphoid/myeloid progenitors primed (LMPPs; CD34^+^CD38^+^CD117^+^), and common lymphoid progenitors (CLPs; CD34^+^CD38^+^CD117^–^CD127^–^) were significantly decreased in transplant recipients as compared with 12 healthy controls (Figure 1A). Figure 1A illustrates the decrease in LMPPs and in CLPs among CD34^+^ bone marrow cells.

Unsupervised clustering, using FlowSOM, according to 44 cell surface and intracellular antigens, disclosed 27 annotated clusters from 24,022,008 cells (Figure 1B) as illustrated in Figure 1C.

### Acute GVHD history discriminates patients with impaired B cell lymphopoiesis.

**Figure 2:**
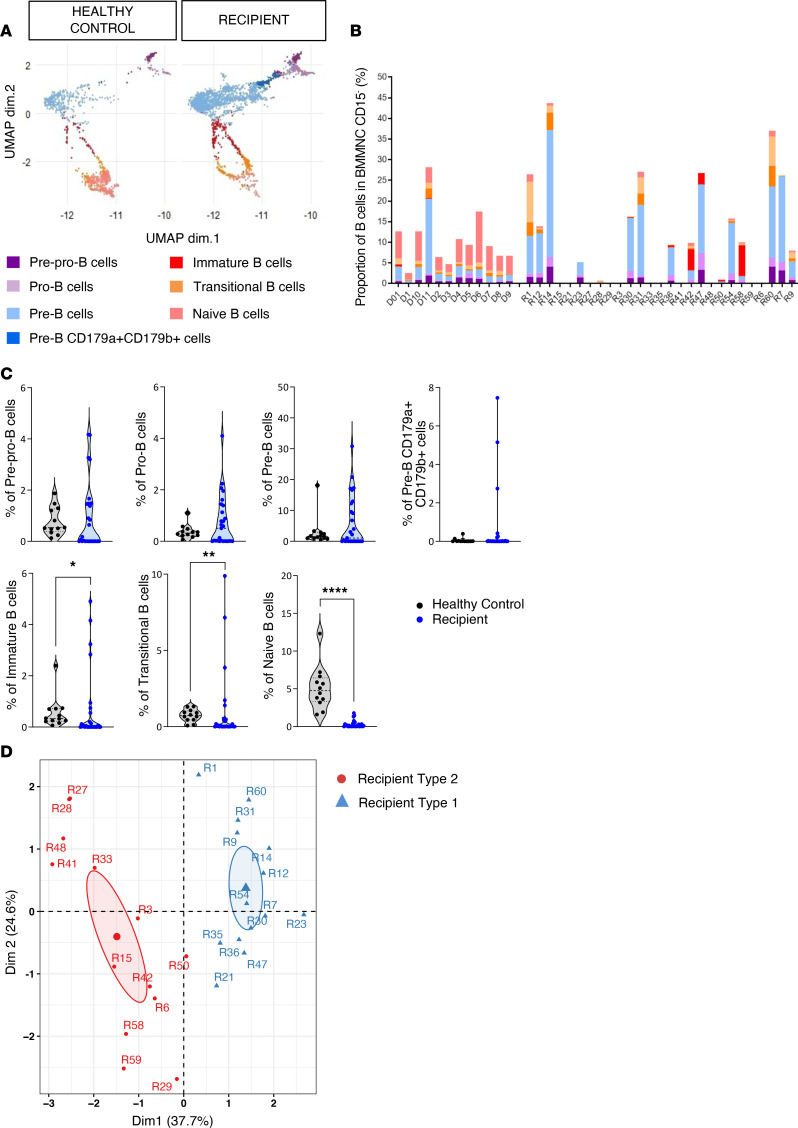
Segregating B cells into phenotypically distinct subsets in human bone marrow. (**A**) UMAP visualization of B cell populations from human CD15^–^ BMMNCs from controls and recipients. B cell populations were defined by FlowSOM in Figure 1B. Colors indicate clusters. (**B**) Data showing the individual cellular compositions of B cell populations per individual. (**C**) Quantification of percentages of all B cell subsets from human CD15^–^ BMMNCs from controls and recipient patients. Mann-Whitney tests: *P < 0.05; **P < 0.01; ****P < 0.0001. (**D**) Principal component analysis (PCA) generated using all markers on B cell population subsets identified in **A**. PCA identified 2 clusters among all recipients. Each point represents a recipient (*n* = 26).

When focusing first on the B cell compartment, we observed a strong interpatient variation in the representation of the different B cell subsets (Figure 2, A and B). Notably, there was a significant decrease in immature, transitional, and naive B cells in patients, while the proportion of more early progenitors was not different between healthy controls and transplant recipients (Figure 2C).

To investigate biological disparities in B cell subsets, we used principal component analysis (PCA) and hierarchical *k*-means based on B cell subset abundances. This separated recipients into 2 distinct groups (hereafter designated as R1 and R2) (Figure 2D and Supplemental Figure 6).

**Figure 3:**
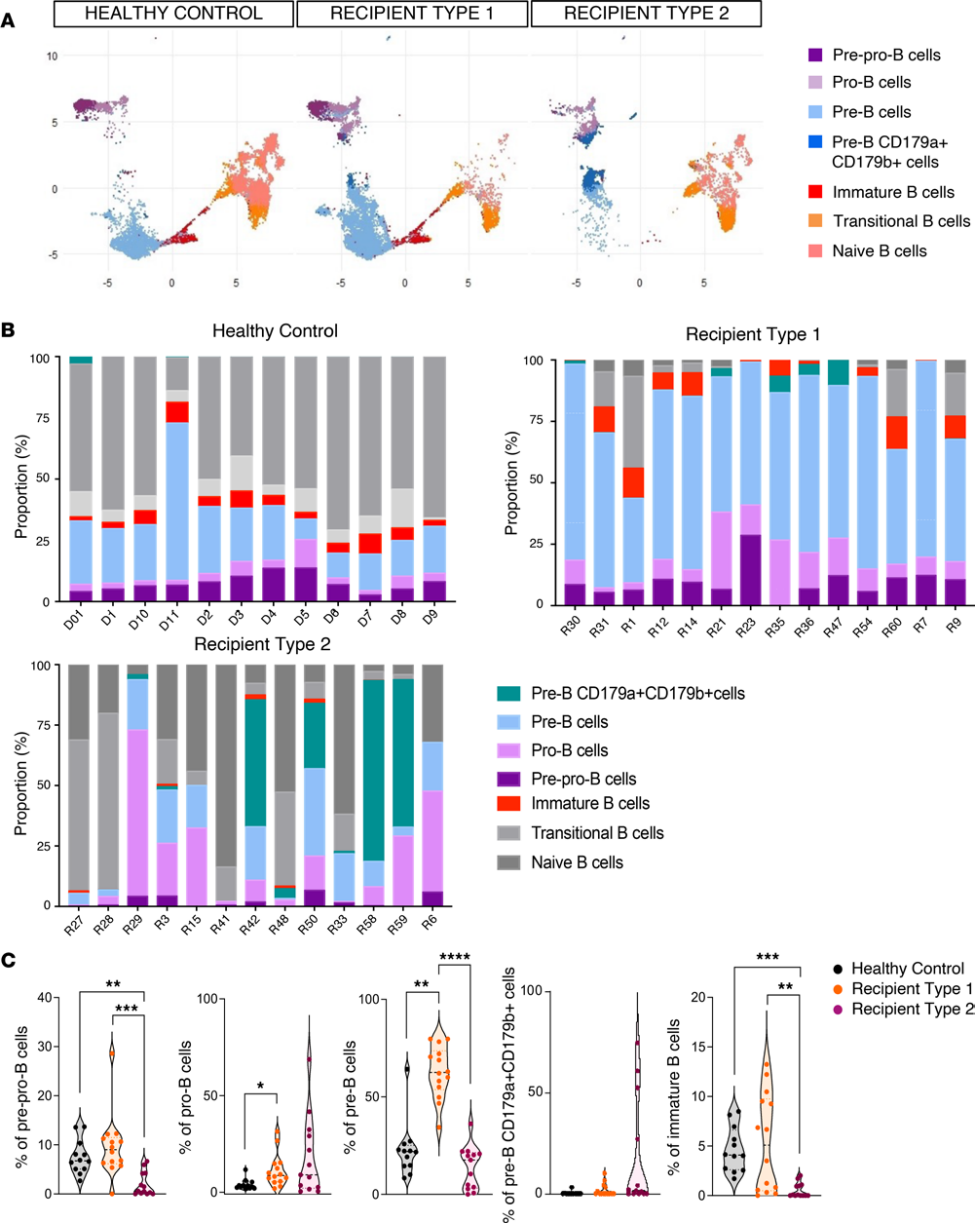
B cell engraftment. (**A**) UMAP visualization of B cell populations from human CD15^–^ BMMNCs from healthy controls (*n* = 12), recipients of group 1 (R1, *n* = 14) and recipients of group 2 (R2, *n* = 12) patients. Colors indicated clusters. (**B**) Data showing the individual cellular compositions of B cell populations in total B cells per individual and per group (controls, R1 and R2 patients). In gray are mature B cells and in color are early B cells (pre-pro-B, pro-B, pre-B, pre-B CD179a^+^CD179b^+^, and immature B) corresponding to populations of interest. (**C**) Quantification of percentage of early B cell populations in controls (black) and R1 (orange) and R2 (pink) recipients. *P* values correspond to Kruskal-Wallis test: *P < 0.05; **P < 0.01; ***P < 0.001; ****P < 0.0001.

When we compared the frequency of B cell subsets in the 2 recipient groups, we found decreased pre-pro-B, pre-B, and immature B cells (and a moderate increase in pro-B) in R2 patients (Figure 3, A and B). R1 patients were uniquely characterized by increased proportion of pre-B cells relative to both healthy controls and R2 patients (Figure 3C). However, despite reduced frequencies of B cell progenitors, mature B cells retained the ability to undergo functional B cell receptor (BCR) rearrangement (Supplemental Figure 7A), although diversity was reduced (Supplemental Figure 7B) in comparison with healthy bone marrow controls. Among tested bone marrow cytokines only BAFF and IL-7 were increased in transplant recipients (Supplemental Figure 7C).

We then searched for clinical factors associated with R1 and R2. As shown in Table 1, only previous acute GVHD (before sampling) significantly discriminated R1 and R2 patients (*P* = 0.012). Among biological peripheral parameters, none were different between R1 and R2, except higher reticulocyte count in R2 (*P* = 0.046) (Supplemental Figure 2).

### Committed myeloid progenitors compensate for the lack of early precursors after allogeneic HSCT.

**Figure 4:**
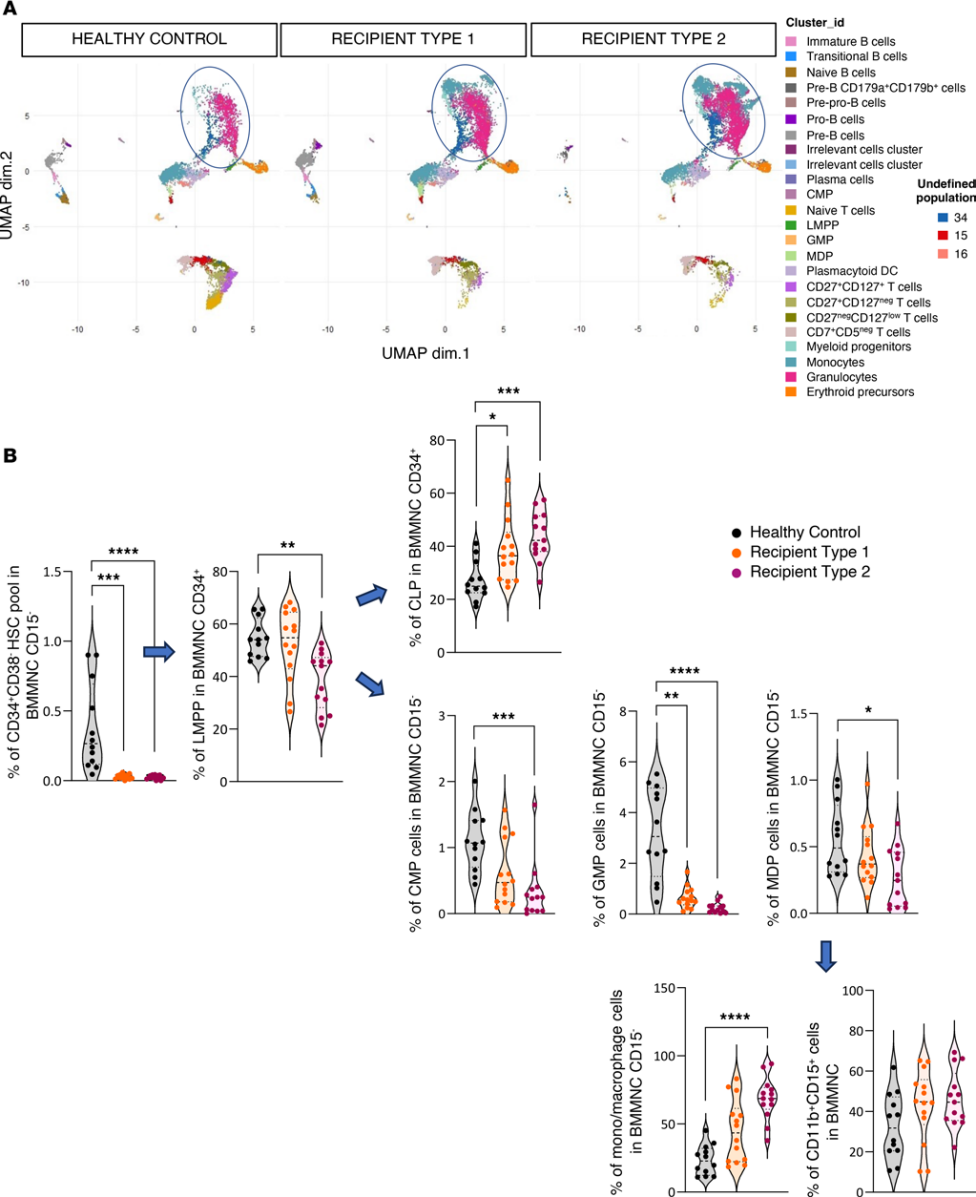
Early progenitors and mature myeloid engraftment. (**A**) UMAP plot generated from an equal subsampling by subset from CD15^–^ BMMNCs from controls (*n* = 12) and recipients (*n* = 26) (44 antigens). Clusters are color-coded, and mature myeloid and early progenitors are circled in blue. (**B**) Quantification of percentage of the HSC pool (CD34^+^CD38^–^) from CD15^–^ BMMNCs; LMPPs and CLPs from CD34^+^CD15^–^ BMMNCs; common myeloid progenitors (CMPs; CD34^+^CD38^+^CD123^+^CD45RA^–^); granulocyte and monocyte progenitors (GMPs; CD34^+^CD38^+^CD123^+^CD45RA^+^); macrophage dendritic cell precursors (MDPs; CD34^lo^CD38^–^CD117^int/lo^CD45RA^–^); and monocytes/macrophages from CD15^–^ BMMNCs and granulocytes from CD11b^+^CD15^+^ BMMNCs in controls and R1 and R2 recipients. *P* values correspond to Kruskal-Wallis test: *P < 0.05; **P < 0.01; ***P < 0.001; ****P < 0.0001.

Seven clusters of early and committed myeloid progenitors were identified with FlowSOM (Figure 1B) and are illustrated in Figure 4A using uniform manifold approximation and projection (UMAP). In the 3 groups (D, R1, and R2), early progenitors (HSCs, LMPPs, and CLPs) and already committed myeloid progenitors (common myeloid progenitors [CMPs] and granulocyte and monocyte progenitors [GMPs]) were scarce. This was expected, as cells already committed to the myeloid lineage account for high numbers of CD15^–^ bone marrow cells (clusters 36 and 35 [myeloid progenitors]: CD38^lo^, CD45^+^, CD10^+^, CD66b^+^, and CD11b^+^), pre-neutrophils (22) (cluster 31 [granulocytes]: CD38^+^, CD24^+^, and CD66b^+^, which account for nearly 25% of total cells), and monocyte precursors (clusters 26, 32, 37, and 38 [monocytes]: CD66b^+^ and CD11b^+^).

We thus used, as described by Bendall and colleagues (21), a gating strategy (Supplemental Figure 5B) to analyze early bone marrow progenitors. This analysis showed that a large proportion of myeloid cells were indeed monocytes/macrophages or granulocytes (CD11b^+^CD15^+^). In sharp contrast with mature cells, the proportion of LMPPs, CMPs, and, to an even greater degree, GMPs was strongly decreased in R1 and R2 (Figure 4B).

Altogether, these data show that the relative abundance of myeloid and B cell engrafted progenitors profoundly differed in patients as compared with healthy controls with a shift toward terminal myeloid differentiation and a decrease of most myeloid and B cell progenitors.

### Bone marrow T cell subsets disclose similar repartition in the 2 recipient groups.

Since R1 and R2 patients significantly differed regarding previous GVHD, we characterized in 8 patients and 6 healthy controls the phenotype of bone marrow T cells using a T cell–oriented antibody panel (*n* = 18 antibody; Supplemental Table 5A) by mass cytometry (phenotypic definitions of cell populations are described in Supplemental Table 5B). FlowSOM algorithm identified 41 clusters that were merged into 27 meta-clusters (Supplemental Figure 8A). Bone marrow T cell subsets were decreased as compared with healthy controls (Supplemental Figure 8B) and varied among subtypes with the strongest decreases in naive, central memory (Tcm), and effector memory (Tem) CD4^+^ T cells and naive CD8^+^ T cells, in recipients as compared with healthy controls (Supplemental Figure 8C). We then studied whether these bone marrow T cell subsets varied according to recipient type R1 (*n* = 4) and R2 (*n* = 4) or expressed different functional markers, as compared with healthy controls (*n* = 6). Results are summarized in Supplemental Figure 9. No strong differences emerged from the analyses of T cell subsets according to R1 and R2, but that might be limited by the number of studied samples (*n* = 4 per group). However, analysis of functional markers disclosed coexpression of PD-1 and TIGIT in naive T cells and CD4^+^ Tcm cells from R1 patients and decreased expression of CD73^+^ in naive CD8^+^ T cells in both patient groups.

Altogether, while our initial aim was to focus on B cell progenitor engraftment, our results clearly illustrated that profound alterations in all B cell and myeloid progenitors characterize engraftment 3 months after HSCT, with previous GVHD being a driver parameter of patients’ heterogeneity. Analyses of T cell subsets also revealed alterations in subsets, some of which associated with markers of T cell exhaustion but irrespective of GVHD. We then decided to go deeper into the underlying mechanisms that could explain these differences by exploring transcriptomics profiles of hematopoietic precursor and immune cells in bone marrow by simultaneous analysis of 137 surface markers and of RNA sequences at the single-cell level using cellular indexing of transcriptomes and epitopes by sequencing (CITE-Seq).

### Single-cell analyses of bone marrow cell populations using CITE-Seq.

Four healthy controls and seven R1 and three R2 recipients were analyzed using CITE-Seq (Supplemental Table 6, A and B). Phenotypic definitions of bone marrow cell populations are summarized in Supplemental Table 6C. A total of 72,566 cells were analyzed (21,958 from healthy controls, 33,615 from R1, and 16,993 from R2 recipients). Roughly, a mean of 5,000 cells were analyzed per patient (5,490 for healthy controls, 5,664 for R1, and 4,802 for R2 patients); after filtering, 20,691 RNA sequences were analyzed.

**Figure 5:**
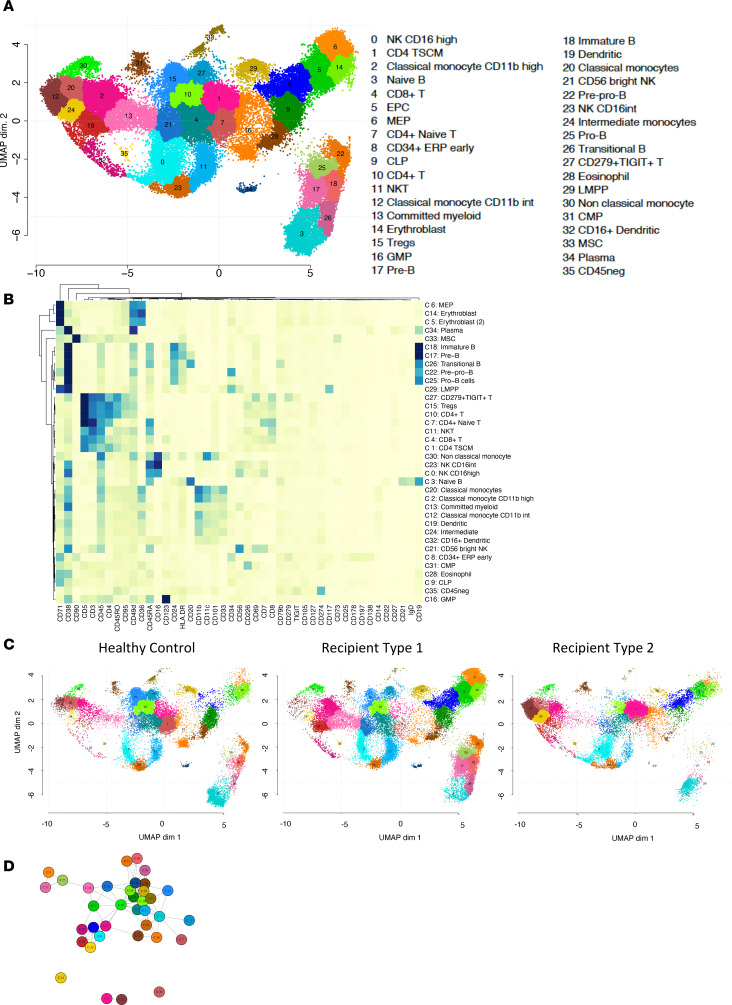
A comprehensive single-cell proteo-genomic map of controls, recipients of group 1, and recipients of group 2. (**A**) UMAP display of single-cell proteo-genomics data by CITE-Seq of human bone marrow from controls and recipient of cluster 1 and recipient of cluster 2 patients (*n* = 72,566 single-cell, 137 surface markers); *n* = 14 samples. Clusters are color-coded, and cell types associated with each cluster are displayed. (**B**) Heatmap representing scaled expression of phenotype antigen across the cell subsets manually ordered and annotated for visualization purposes. (**C**) UMAP visualization from controls (*n* = 4) and recipient cluster 1 (*n* = 7) and recipient cluster 2 (*n* = 3) patients. (**D**) Connection graph of clusters. Two clusters are connected if their relative abundance is not significantly different between R1 and R2 recipients (see Methods). Four classes of cluster appear (clusters 24, 1, 12, and 20), with no connection between them: these clusters differentiate R1 and R2 recipients.

**Figure 6:**
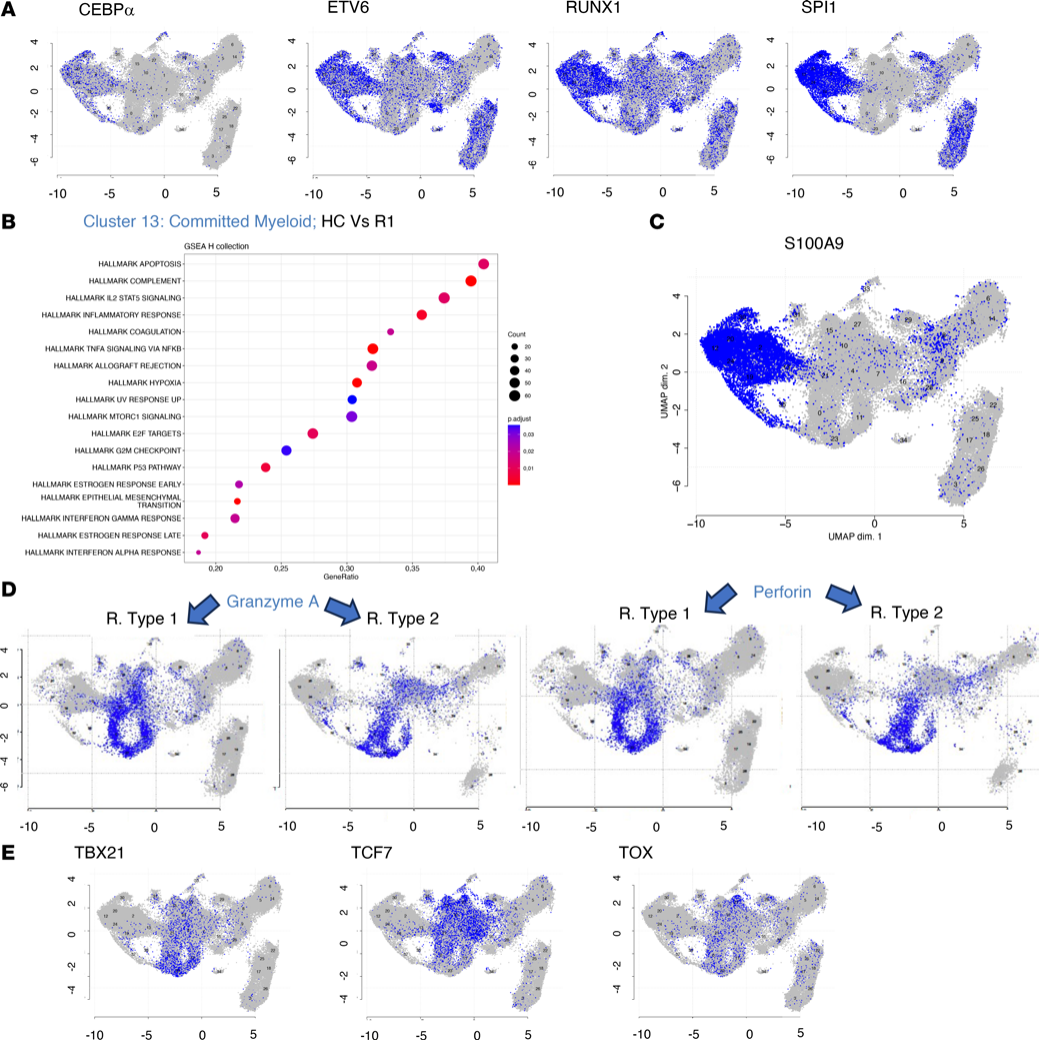
Gene expression of selected genes. (**A**) Expression of selected mRNAs highlighted on the UMAP from Figure 5A. Expression of several mRNAs expressed by myeloid-committed progenitors. (**B**) Functional enrichment analysis of annotated genes using hallmark collection. GSEA analysis based on log fold change from limma-trend analysis on scran-normalized data. The figure shows significant (Benjamini-Hochberg, adjusted *P* values < 5%) functional enrichment in biological states or processes analysis in committed myeloid progenitors (cluster 13). (**C**) RNA expression of S100A9 in the UMAP from Figure 5A. (**D**) RNA expression of granzyme A and perforin in the UMAP from R1 and R2 recipients. (**E**) Expression of selected mRNAs highlighted on the UMAP from Figure 5A. Expression of TBX21, TCF7, and TOX by T cells.

After harmonization (see Methods), 36 clusters were segregated according to cell surface antigen expression using Seurat and visualized using UMAP (Figure 5A). A heatmap of selected phenotypic markers that allowed cell population assignment is shown in Figure 5B. Projection of R1 and R2 patients into this UMAP showed segregation of the 2 patient groups (Figure 5C). This difference in cluster abundances between R1 and R2 recipients was statistically significantly different in compositional analysis as illustrated in a disjoined graph (Figure 5D). Finally, we checked that canonical transcription factors described in the literature projected as expected on phenotypically defined populations (Figure 6A and Supplemental Figure 10).

We then analyzed results according to their cell surface phenotypes and grouped clusters into meta-populations of physiological relevance.

### Bone marrow B cell subsets.

Six populations (clusters 22 [pre-pro-B], 25 [pro-B], 17 [pre-B], 18 [immature B], 26 [transitional B], and 3 [naive B]) were found to segregate in a B cell meta-cluster. As expected, all expressed *EBF1*, *PAX5*, *TCF3*, and *SPI1*, while *RAG1* and *RAG2* were not expressed in transitional and naive B cells (Figure 6A and Supplemental Figure 10). In agreement with mass cytometry data, early B cell precursors (pre-pro-B, pro-B, pre-B, and immature B cells) were largely underrepresented in R2 recipients, who basically had only detectable naive B cells (Figure 5C).

Gene set enrichment analysis (GSEA) disclosed hallmarks of inflammation and TNF-α signaling via NF-κB in pre-pro-B cells when controls were compared with R1. In R2 recipients, naive B cells disclosed hallmarks of the IFN-α response, TNF-α signaling via NF-κB, and allograft rejection signature (Supplemental Figure 11, A and B). A single small cluster was assigned to plasma cells in healthy controls and R1 patients (cluster 34) sharing hallmarks of allograft rejection, TNF-α signaling, and IFN-α response. Notably, as compared with healthy controls, R1 recipients disclosed hallmarks of inflammatory and of IFN-α and -γ responses. In addition to these 3 hallmarks, that of allograft rejection signature was also found in R2 recipients (Supplemental Figure 11B).

### Lymphoid and myeloid progenitors.

Four populations of early progenitors (9 CLP, 29 LMPP, 31 CMP, and 16 GMP) were identified. Myeloid progenitors expressed *RUNX1* and *SPI1*, while CLP expressed *EBF1*, *PAX5*, and *SPI1*, and LMPP expressed *SPI1* and *RUNX1*. All populations expressed TGF-β and TGF-β receptors 1 and 2 (IL-18 and TNF-α were also expressed by GMP) (data not shown). Lymphoid progenitors (CLP and LMPP) had hallmarks of IL-2/Stat5 pathways in both recipient groups. All progenitors in these groups shared hallmarks of inflammation, TNF-α signaling, and allograft rejection (Supplemental Figure 11C).

A population of already committed myeloid progenitors (cluster 13) was found in all patients and healthy control groups. Its phenotype was CD34^lo^CD38^hi^CD11b^+^, CD33^+^, CD16^–^ and had the following transcription factor profile: CEBPα, ETV6, RUNX1, and SPI1. Hallmarks in cluster 13 were associated with inflammation, allograft rejection, TNF-α signaling via NF-κB, and INF-α and IFN-γ signaling (Figure 6B). Notably, both monocytes and committed myeloid progenitors highly expressed protein of the *S100* family, recently linked to bone marrow GVHD in single-cell analyses (23) (Figure 6C).

### Inflammatory environment characterizes monocyte subsets after allogeneic HSCT.

Five populations of monocytes were segregated (clusters 2, 12, 20 [classical], 24 [intermediate], and 30 [non-classical]; see Supplemental Table 6C for phenotypic definition) and 2 populations of myeloid dendritic cells (DCs) (clusters 19 [classical DC1] and 32 [monocyte-derived DC/classical DC2]). R2 patients disclosed significant difference in 2 classical monocyte clusters (clusters 12 and 20) and 1 intermediate monocyte cluster (cluster 24) (Figure 5D). Monocyte- and DC-associated hallmarks are illustrated in Supplemental Figure 11D, showing that in comparison with healthy controls, bone marrow monocytes from transplant recipients had hallmarks of inflammatory response and IL-2/Stat5 and INF-α and -γ signaling (cluster 30). Myeloid DCs (cluster 19 and 32) disclosed hallmarks of several pathways associated with inflammation and allograft rejection.

### Erythroid/megakaryocytic progenitors and erythroblasts.

The meta-cluster of erythroid/megakaryocytic progenitors and erythroblasts included clusters 6 (megakaryocyte erythroid progenitors [MEPs]), 8 (CD34^+^ early erythroid progenitors [ERPs]), 5, and 14 (erythroblast). Cluster 6 (MEP) was mostly represented in R1. Cluster 8 (early ERP) was represented only in R2 recipients who disclosed TNF-α and inflammation hallmarks. Cluster 14 (erythroblast) exhibited hallmarks of TNF-α and IFN-α and -γ signaling (Supplemental Figure 12).

### Bone marrow NK, NKT, and T cells exhibit an activated and inflammatory profile.

Three NK clusters (0, 21, and 23) and one NKT (11) were segregated. As compared with healthy controls, increased expression of granzyme and perforin and upregulation of HLA class I molecules were disclosed in R1 patients. All NK clusters disclosed hallmarks of IFN-α and -γ response and of allograft rejection. The NKT cluster exhibited hallmarks of TNF-α signaling, allograft rejection, and apoptosis (Supplemental Figure 13).

Finally, 6 clusters were included in the meta-cluster of T cells. Cluster 1 of CD4^+^ T cells was highly represented in R2 recipients. Phenotypically, it expressed TCRαβ, CD194, CD25, CD27, CD28, CD95, CD134, and CD183 (CXCR3) and was negative or low for CD62L, CD183, and CD127. This extended phenotype is compatible with stem cell memory T cells. Other T cell clusters included cluster 15, CD4-exhausted Tregs (*T-BET*, *TCF7*, *TOX*, *LAG3*, and *TIGIT*), which was underrepresented in R2; cluster 10 (CD4 effector memory, expressing TCF7); cluster 7 (naive CD4^+^ cells expressing *T-BET* [*TBX21*] and *TCF*); cluster 27 (CD4-exhausted memory, expressing *TOX*, *LAG3*, and *TIGIT*); and one cluster (cluster 4; CD8-naive) with exhaustion features (expressing *TOX* and *TCF7*, as well as *LAG3*, *TIGIT*, and *PD1*) (Figure 6E). Functionally, T cell subsets expressed markers of cytotoxicity (granzyme A [*GZMA*], *GZMK*, perforin [*PRF1*], *TGFβ*, *TGFβ-R2*, and *TGFβ-R3*) (Figure 6D).

Comparing controls with R1 using GSEA hallmarks in these T cell clusters showed hallmarks of TNF-α signaling, with strong expression of perforin, *GZMA*, *GZMB*, and *GZMK*.

### Mesenchymal stem cells.

A small cluster (cluster 33) compatible with a mesenchymal stem cell phenotype (CD45^–^CD34^–^CD105^+^CD90^+^CD73^+^) was represented in healthy controls and R1 patients and showed increased hallmarks of allograft rejection and epithelial-mesenchymal transition as well as of IFN-α in patients, suggesting an alteration of bone marrow stromal environment induced by inflammation after allogeneic HSCT.

**Figure 7:**
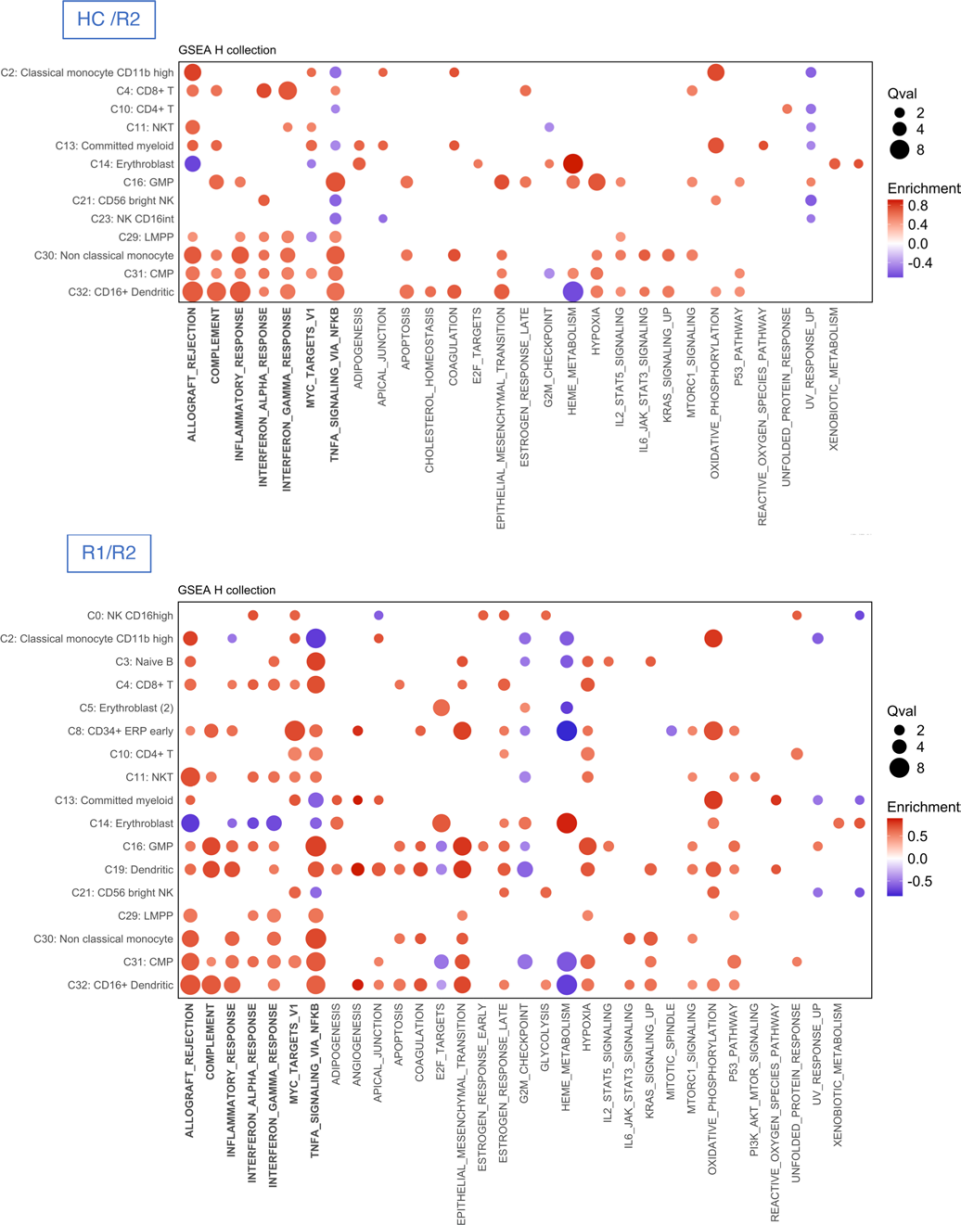
Comparison of gene signatures between controls and recipients of group 1 and group 2. Dot plots depicting enrichment analysis within each cluster of cells with the use of hallmark gene sets in healthy control (HC) versus recipient (R) groups. All significant pathways (Benjamini-Hochberg, adjusted *P* values < 5%) from GSEA analysis are presented as dots whose sizes correspond to the *q* values [–log_10_(*q*)] and colors to the enrichment score. Comparisons between HCs and recipients of group 2 (R2) and between recipients R1 and R2 are illustrated.

To sum up, CITE-Seq analyses revealed a profound alteration in cell subset distribution between healthy controls and R1 and R2 recipients. T cell and NK cell clusters were characterized by *TCF7* expression, but dual expression of *TOX* and *TCF7* (as marker of exhaustion) was mainly limited to clusters 10, 21, 27, and 34 (Supplemental Figure 14A). Single-cell RNA-Seq analyses suggest a potent role of TNF-α and IFN-γ on myeloid progenitors. TNF-α was mainly expressed in monocytes and committed myeloid progenitors, IFN-γ in NK cells, and IFN-γ receptors in committed myeloid progenitors, monocytes, and B cell progenitors (Supplemental Figure 14B). Finally, GSEA hallmarks repeatedly disclosed a similar pathway in transplant recipients, namely: allograft rejection, complement, inflammatory response, TNF-α signaling via NF-κB, IFN-α and -γ response, and Myc targets (Supplemental Figure 15 for healthy controls vs. R1, and Figure 7 for healthy controls vs. R2 and R1 vs. R2).

A summary figure of combined cytometry by time of flight (CyTOF) and CITE-Seq data is provided as Supplemental Figure 16.

## Discussion

Engraftment is a crucial step for successful allogeneic HSCT. However, the interplay of the different bone marrow cell subsets implicated in this process has seldom, if ever, been described in humans. In this study, we used both mass cytometry and CITE-Seq to uncover the complexity of this process among all bone marrow cell populations. We aimed first to underlay the origin of the early post-transplantation bone marrow B cell defect. Analyses of the bone marrow cell populations using mass cytometry revealed that B cell progenitors’ defect was associated both with alterations of myeloid cell precursors and with NK and T cell activation. These modifications were profoundly associated with the occurrence of previous GVHD, and inflammation as illustrated by single-cell proteo-transcriptomics analyses.

Much of our understanding of stem cell dynamics in transplantation comes from experiments in experimental model organisms, based on clone-tracking methods that “barcode” the genomes of endogenous or transplanted HSCs. However, direct human studies are limited by a paucity of applicable methodologies. One exception is the tracking of vector integration sites. Gene therapy trials provided estimates of engrafting HSC numbers in this autologous setting (8, 9, 24, 25). However, we are not aware of detailed analyses in the allogeneic setting, except that of Montaldo et al. (as discussed below) (26).

As previously described in the literature, early reconstitution of B cells after allogeneic HSCT is evidenced by a shift in peripheral blood toward transitional and naive B cells with almost no memory B cells until 1 year after transplantation (reviewed in ref. 11). However, bone marrow engraftment of B cell progenitors has been poorly characterized so far. After experimental transplantation, it has been reported that alloreactive Fas^+^ T cells can target the HSC niche, inducing impaired B-lymphoid differentiation from HSCs (27), and more recently that B cell lymphopoiesis is impaired in Treg-depleted mice (28). In the human setting, one study (29) found greater numbers of B cells expressing PAX5 in the bone marrow of patients who did not develop chronic GVHD (as compared with patients who did). In another study by Mensen et al. (30), decreased peripheral B cell reconstitution was associated with increased T cell infiltration in bone marrow biopsy, which was associated with reduced numbers of osteoblasts, suggesting that GVHD can target bone marrow stromal cells and impair B cell lymphopoiesis. Here, we deciphered interpatient variability in B cell progenitors by identifying 2 distinct groups of recipients using PCA. Clinically, these 2 groups of patients differed only in prevalence of GVHD, which was significantly more frequent in the R2 recipients. R2 recipients had decreased pre-pro-B, pro-B, and immature B cells, while R1 were characterized by increased abundance of pre-B cells relative to both healthy controls and R2 recipients. Despite decreased frequency (as compared with healthy controls) and the heterogeneity of precursors in the 2 patient groups, some bone marrow B cell progenitors retained their ability to fully rearrange their BCR, leading to an oligoclonal pattern.

We then decided to extend our analysis to lymphoid or myeloid engraftment. We uncovered that there was an increase in the proportion of monocytes/macrophages and a similar proportion of granulocytes, as compared with healthy controls. In sharp contrast to mature cells, the proportion of LMPPs, CMPs, and GMPs was strongly decreased. In both patient groups, bone marrow T cells were decreased in comparison with healthy controls. We also observed variations in T cell subtypes, with a marked decrease in naive CD4^+^ and CD8^+^ T cells, some of which disclosed features of exhaustion.

Although mass cytometry has previously been used to describe peripheral blood hematopoietic reconstitution in humans after allogeneic HSCT (31, 32) and 2 seminal papers used CyTOF to decipher the development of B cells within the bone marrow (20, 21), we are not aware of mass cytometry studies aiming at deciphering B cell progenitor and myeloid engraftment in humans.

We then aimed to capture biological mechanisms through CITE-Seq analyses. We analyzed 10 patients and 4 healthy controls. In allogeneic recipients (especially in R2) sustained engraftment was shifted toward committed erythroid and myeloid progenitors, suggestive of emergency hematopoiesis as reported by Montaldo et al. (26). Since R2 recipients are characterized by increased frequency of GVHD, these findings might be of special interest to decipher a prevalent (but poorly understood) clinical problem, i.e., “poor marrow function” or “late graft failure” (33–37). Indeed, bone marrow GVHD was experimentally described 14 years ago (27), but the bona fide effect on bone marrow cell populations is basically unknown. Furthermore, since committed myeloid progenitors were dominant in transplant recipients, this may explain why additional bone marrow insult by viruses (CMV or human herpesvirus 6), Gram-negative bacteria, or drug-mediated toxicity often leads to profound cytopenia in these patients. From a more basic point of view, these results also raise the question of which bone marrow cell populations lead to initial sustained engraftment. Although gene insertion tracking has already suggested that short-term repopulating cells are mainly driving hematopoiesis early after gene therapy (8, 9), recent evidence in nonhuman primates suggests that true HSCs can lead to early neutrophil recovery (38). No clonal marker could be used in the setting of allogeneic HSCT. However, our results suggest that committed myeloid progenitors may be short-term repopulating cells. This, of course, does not preclude that HSCs did not engraft in these patients, who are now surviving more than 2 years after transplant and have normal blood counts.

Detailed analyses of the 36 clusters grouped into 5 main subsets allowed in-depth discrimination of cellular populations in transplant recipients fitting with distribution of bone marrow cell populations in previous studies in healthy controls (5, 8). However, because of the rarity of HSCs in CD15^–^ BMMNCs, the in-depth characterization of HSCs was not possible. Furthermore, contrasting with experimental data on Treg and B lymphopoiesis (28), it should be noted that bone marrow Tregs (CD4^+^, CD25^+^, Foxp3^+^) in our analyses accounted for only 15% of human CD4^+^ T cells as compared with nearly 40% of CD4^+^ in mouse bone marrow (39).

In mice, Xie et al. analyzed emergency hematopoiesis in the setting of infection (40). In a previous study, Montaldo et al. (26) performed a comprehensive immunophenotypic and transcriptome analysis, at a bulk and single-cell level, of neutrophils from healthy donors and patients undergoing stress myelopoiesis. This study included exposure to growth factors, pancreatic cancer, and viral infection but also some patients who underwent allogeneic HSCT (mostly in blood but also including some with bone marrow study). This study, mostly focused on neutrophil generation, provided evidence of emergency hematopoiesis linked to interferon in the setting of allogeneic HSCT. Here CD15^+^ cells were depleted in CITE-Seq experiments and thus did not address neutrophil recovery. We provide additional data supporting the role of acute GVHD through detailed analysis of T and NK cell subsets. More recently, one study dissected human hematopoietic reconstitution after allogeneic HSCT (23). The authors sequentially analyzed 10 patients with severe aplastic anemia (SAA) and used single-cell RNA-Seq of Lin^–^CD34^+^ sorted bone marrow cells. The authors paid much attention to residual recipient cells, as mixed chimerism is common and raises concerns in patients with SAA because of the risk of rejection (41). However, in patients with acute myeloid leukemia, mixed chimerism is associated with an increased risk of relapse, which was not a concern herein (1 patient studied with CyTOF; none of those studied by CITE-Seq). Finally, Huo et al. found that neutrophil progenitors express low levels of the S100A gene family in patients who subsequently develop GVHD (23). Although we did find increased expression of S100 protein in myeloid progenitors, we did not find differences in R2 recipients (who more frequently had GVHD) as compared with R1 patients. This suggests that S100 proteins in human engrafted marrow could play a role as alarmins in a highly inflammatory environment (42), regardless of GVHD.

Finally, we compared the transcriptional programs in the different cellular populations between healthy controls and the 2 patient groups. Enrichment analyses (GSEA) repeatedly showed that BMMNCs from transplanted patients, compared with healthy controls, disclosed hallmarks of TNF-α signaling via NF-κB, IFN-γ response, and allograft rejection, which in our setting reflects the allogeneic reaction of donor cells against the recipient. Furthermore, IL-1β and TGF-β were mostly expressed in transplant recipients. TNF-α and IL-1β are major determinants of aged and stressed hematopoiesis (43–51), and thus these cells might be considered as having both an emergency hematopoiesis and a highly inflammatory environment generated by the allogeneic reaction. IFN-γ is a major mediator of immune-mediated bone marrow failure both in the setting of acquired aplastic anemia (41, 52–54) and in cytopenia developing after chimeric antigen receptor T cell therapy (55), and an interferon signature has already been reported in allogeneic transplant recipients by Montaldo et al. (26). A recent study using spatial transcriptomics also disclosed the same hallmarks in gastrointestinal tissue affected with GVHD (56). Thus, we suggest that T cells (and NK cells) are major drivers of the observed perturbation in the distribution of cellular populations observed at engraftment. Previous experimental and clinical studies of bone marrow T cell depletion support this hypothesis (57–61). However, T cells not only promote engraftment but also drive graft–versus–bone marrow effects including the graft-versus-leukemia effect and T cell–mediated damage to the bone marrow environment (62, 30). Furthermore, patients with GVHD were treated with high-dose corticosteroids, and patients with steroid-resistant GVHD were also treated with the JAK1/2 inhibitor ruxolitinib. The relative contribution of these interacting factors cannot be discriminated in the present analyses.

While being a quite unique study of human bone marrow engraftment, this study has limitations. The mass cytometry antibody panel was designed to decipher B cell progenitors and thus had less coverage on myeloid progenitors and on T cell phenotype. This study examined bone marrow at a clinically relevant point but is not longitudinal. Single-cell CITE-Seq experiments have been performed on CD15^–^ cells but did not aim to sort purified HSCs. Finally, among others, it would be worth exploring TNF-α and IFN-γ responses in conditional-knockout mouse transplantation models to dissect their effect on bone marrow hematopoiesis.

In conclusion, this study provides evidence that B cell recovery and myeloid reconstitution are severely impaired mainly by T cell–mediated processes. Similar approaches could be applied, for example, to decipher the poorly studied interactions between myeloid malignancies and the immune system, in human bone marrow samples.

## Methods

### Sex as a biological variable

Patients and healthy controls were of both sexes.

### Human biological samples

Patients with myeloid malignancies underwent allogeneic HSCT at the Hospital Saint Louis, Paris, France. Bone marrow was also harvested from healthy controls in this cohort during bone marrow donation. However, the healthy controls were unrelated to the patients. For the second cohort, patients were included just before transplantation with a blood sample at 0, 3, and 6 months and a bone marrow sample at 3 months. Healthy controls in this cohort agreed to donate a blood sample for research as part of blood donation. The inclusion criterion was adult patients (18 years or older). Patients who received anti-CD20 (rituximab) for EBV reactivation after transplantation were excluded.

### Cell processing

PBMCs and bone marrow mononuclear cells (BMMNCs) from recipients and healthy controls were isolated by density gradient centrifugation (Pancoll human, density 1.077 g/mL, Pan Biotech, catalog P04-60500) within 3 hours after blood and/or bone marrow sampling and either used immediately for mass cytometry or cryopreserved in liquid nitrogen in heat-inactivated fetal calf serum with 10% DMSO (Sigma-Aldrich, catalog 67-68-5).

### Metal-labeled antibody staining

All metal-labeled antibodies used in this study are summarized in Supplemental Table 4A; 29 prelabeled antibodies were from Fluidigm, and 16 unlabeled antibodies (BioLegend and eBioscience) were conjugated with metal isotopes in our laboratory. Maxpar X8 antibody Labeling Kits (Fluidigm) were used for lanthanide labeling, following the manufacturer’s instructions. In this case, the metal-conjugated antibodies were retrieved in Ab stabilizer PBS (Candor Bioscience) and stored at 4°C until ready for experiments. Maxpar MCP9 Antibody Labeling Kits (Fluidigm) were used for cadmium labeling, and the metal-conjugated antibodies were retrieved in HRP-Protector (Candor Bioscience) at 4°C.

After density gradient centrifugation, the PBMC and BMMNC samples were incubated with Pierce universal nuclease (25 U/mL; Thermo Fisher Scientific, catalog 88702) for cell lysis for 30 minutes at 37°C. Cells were washed twice with RPMI medium, and 10 million cells were incubated with Cell-ID cisplatin (2.5 μM; Fluidigm, catalog 201064) for 5 minutes at room temperature. After washing, cells were resuspended in Maxpar Cell Staining Buffer (Fluidigm, catalog 201068) and Fc Receptor Blocking Solution (BioLegend, catalog 422302) and subjected to a first antibody staining for 30 minutes at 37°C. Then, a second antibody staining was added for 30 minutes at 4°C. After washing twice, cells were fixed in 2% paraformaldehyde (PFA) in PBS 1× for 15 minutes at room temperature. Then, cells were washed by addition of Permeabilization Buffer 1× (eBioscience, catalog 00-8333-56). After washing, cells were resuspended in Permeabilization Buffer 1×, and intracellular staining was performed for 1 hour at 4°C with indicated antibodies. Cells were washed twice in Permeabilization Buffer, then incubated overnight at 4°C in 2% PFA and 1:6,000 Cell-ID Intercalator-Iridium (Fluidigm, catalog 201192B) and stored at –80°C until the day of acquisition.

### Mass cytometry acquisition

Cells were thawed and resuspended in Maxpar Water (Fluidigm, catalog 201241) at 10^6^ cells/mL with 4-Element EQ Beads (Fluidigm, catalog 201078) before mass cytometry acquisition. Then cells were filtered using a cell strainer cap with 35 μm pores (BD Biosciences). Cell events (10 million) were acquired on the HELIOS mass cytometer (Fluidigm) and CyTOF software version 6.7.1014 (Fluidigm) at the Plateforme de Cytométrie de la Pitié-Salpétrière (CyPS). The maximum of events was acquired to allow identification of rare subsets.

### Mass cytometry data analyses

To avoid batch effects, Flow Cytometry Standard (FCS) files were normalized using the beads-based procedure (with the *premessa* R package). After removal of beads, single cells were gated on iridium (^191^Ir and ^193^Ir) DNA staining, and live leukocytes were gated based on CD45^+^ cisplatin-negative staining. Then, we excluded CD15^+^ cells for PBMC and BMMNC analyses. We used the CATALYST R package to cluster cells with FlowSOM (http://www.r-project.org http://dambi.ugent.be) to identify main populations based on the 44 cell lineage–defining markers for BMMNCs (Supplemental Table 4A); based on the 35 cell lineage–defining markers for PBMCs (Supplemental Table 4A, panel PBMC); and based on the 28 cell lineage–defining markers for T cells (Supplemental Table 5A). After using FlowSOM, we extracted cell populations’ abundances. Uniform manifold approximation and projection (UMAP) was used to plot the B cell populations, total BMMNCs, and total PBMCs and visualize expression of selected markers of interest. Mass cytometry data analyses were performed using FlowJo (version 10.8.1, Tree Star) and the Cytobank platform (Beckman Coulter) as well as R (version 4.0.2) and RStudio (version 1.1.4).

Comparisons between study controls and recipient groups were performed using the Mann-Whitney test. The Kruskal-Wallis test was used to compare 3 groups of patients within the same cell population, followed by Dunn’s post hoc multiple-comparison test. Comparisons of patients’ characteristics were performed by Fisher’s exact or χ^2^ tests. *P* values of less than 0.05 were considered significant. Statistical analyses were performed using R (version 4.0.2) or Prism (version 9, GraphPad Software). PCA was conducted with the FactoMineR R package. Only components that cumulatively explained 60% of variance were kept.

### Immunoglobulin gene sequencing

Next-generation sequencing (NGS) of immunoglobulin (IG) gene rearrangements was used to analyze the BCR repertoire in bone marrow samples from 11 healthy controls and 14 recipients. DNA was extracted from cryopreserved BMMNCs using Maxwell RSC Cell DNA Purification Kit (Promega). An amplicon-based NGS approach was used for sequencing of IGH, IGK, and IGL rearrangements. For the preparation of libraries, 100 ng DNA was subjected to first-step multiplex PCR (one multiplex PCR for each locus), using protocol and primers derived from the BIOMED-2 study (63), modified with the addition of Illumina overhang adapters. A fixed concentration of DNA from leukemia cell lines harboring IG clonal rearrangements was spiked in each PCR as calibrators and quality controls. A second-step PCR was performed to add Illumina adapters and barcodes. Libraries were sequenced on Illumina MiSeq platform using 2 × 250 bp sequencing. A median of 94,127 total reads were obtained per sample. FASTQ files were processed and analyzed using the Vidjil tool (http://www.vidjil.org) (64), an algorithm that gathers reads into clonotypes according to their V(D)J junctions. The number of clonotypes and the Shannon index were calculated to evaluate the frequency and diversity of B cells in bone marrow samples.

### Luminex

ProcartaPlex Human Mix&Match 13-plex Panels kit (Thermo Fisher Scientific, catalog PPX-13-MXXGTEG) was used to quantify APRIL, BAFF, CD40L, IFN-γ, IL-1β, IL-21, IL-22, IL-6, IL-7, LAG-3, perforin, SDF-1α, and TNF-α cytokines in plasma samples from bone marrow following the manufacturer’s instructions. After collection of the plasma fraction, samples were stored at –80°C. All samples were run in duplicate in a single plate. Briefly, we added the Capture Bead Mix to each well of the plate, then placed the plate on the Hand-Held Magnetic Plate Washer for 2 minutes. Next, plates were washed twice with a wash buffer before addition of Universal Assay Buffer and samples followed by 2 hours of incubation on a shaker at 600 rpm. Plates were washed twice; then we added Biotinylated Detection Antibody Mix (Thermo Fisher Scientific) to the plate followed by 30 minutes of incubation on a shaker at 600 rpm. After 2 washings, we added streptavidin-phycoerythrin to the plate and incubated for 30 minutes with shaking. Plates were washed as above, and reading buffer was added for 5 minutes with shaking; then the plate was placed in the Luminex MAGPIX instrument to run. Each sample was measured in duplicate.

### ELISA

TNF-α and IL-6 levels were assayed using Quantikine High Sensitivity ELISA Kits (R&D Systems), and BAFF levels were assayed using Quantikine ELISA kits (R&D Systems), according to the manufacturer’s protocol. All samples were run in duplicate.

### Single-cell RNA-Seq sample preparation

BMMNC samples were thawed rapidly in prewarmed RPMI medium with FCS (1:1 vol/vol), washed, and incubated with Pierce Universal Nuclease (25 U/mL; Thermo Fisher Scientific, catalog 88702) for cell lysis for 30 minutes at 37°C. Cells were washed twice with RPMI medium and resuspended in buffer (PBS, 0.5% BSA, and 2 mM EDTA). Then, samples were depleted of myeloid cells from bone marrow using CD15 MicroBeads (Miltenyi Biotec, catalog 130-046-601) procedure. After depletion, cells were labeled with TotalSeq-C Universal cocktails (Supplemental Table 6A) and additional antibodies (Supplemental Table 6B) following the manufacturer’s instructions (BioLegend) for 30 minutes at 4°C. Then cells were washed with staining buffer and filtered using a 40 μm Flowmi Cell Strainer (Sigma-Aldrich, catalog BAH136800040-50EA). The cells were then counted, and 20,000 cells per sample were combined with barcoded single-cell VDJ 5′ gel beads, master mix, and partitioning oil to generate gel beads emulsion using 10x Genomics Chromium Controller according to the manufacturer’s guidelines. Chromium Next GEM Single Cell 5′ Kits v2 (10x Genomics, catalog PN-1000263) and 5′ Feature Barcode Kit (10x Genomics, catalog PN-1000256) were used to prepare reagents.

The concentration of each sample was measured using TapeStation 2200 (Agilent) to perform single-cell RNA-Seq after cDNA amplification. To prepare the cDNA libraries for 10x Genomics Chromium Controller, we used the single-cell 5′ v2 (Dual Index) with Feature Barcode technology for Cell Surface Protein and 5′ Gene Expression, following the manufacturer’s instructions. Quality control libraries were analyzed using TapeStation 2200 (Agilent). Libraries were equimolar-pooled to obtain at least 40,000 read pairs cell for 5′ Gene Expression and 10,000 read pairs for Cell Surface Protein after sequencing on Illumina NovaSeq 6000 S4 Reagent Kit (200 cycles). The input number of cells was estimated at 10,000 cells per sample. FASTQ files were obtained with bcl2fastq (Illumina).

### CITE-Seq single-cell RNA sequencing acquisition and preanalysis

The 10x Cell Ranger package (version 6.1.2, 10x Genomics) was used to process transcript, CITE-Seq–like alignment, filtering, barcode counting, and unique molecular identifier (UMI) counting using GRCh38 genome assembly as reference data. Then, single-cell RNA-Seq data analyses were performed using the package Seurat (version 4.0), R (version 4.1.1), and RStudio (version 4.1.3), including for graph-based clustering and visualizations.

#### Droplets filtering.

Filtering was done separately for each sample. The following droplets were removed: droplets with 0 total RNA count (empty droplets); droplets with no mitochondrial RNA; droplets with less than 300 RNA (total count, not containing a cell); droplets with more than 10,000 RNA (total count, droplets with 2 or more cells); and droplets with more than 10% of RNA from mitochondrial genes (assuming they contained dead cells).

The results for all patients were then merged before further analysis. Last, transcripts that were found at most one time in each cell were filtered out. The resulting data were used in all further analysis. Surface marker data of Igκ and Igλ were also excluded from analysis because their expression profile suggested a very high nonspecific fixation.

#### CITE-Seq data and cluster analyses.

Droplets were first clustered based solely on the surface marker data. These data were normalized using a centered log-ratio (clr) transformation, with 0 data replaced by 0.5 (“half the quantification limit,” assuming a quantification limit of 1 count; this was necessary for 7.6% of the whole data set, including all markers for all cells of all patients), and then log-transformed.

Quantification data were then harmonized to correct for batch effect and for between-patients variability; each marker was corrected separately. Standard harmonization tools could not be used, first because we generated surface marker data, and second because patients were nested into groups. Hence, raw correction of patient effects would also remove any difference between groups, precluding any differential analysis between groups. Patient effects were removed so that mean marker expression of marker *m* for patient *i* was equal to the mean of this marker in the patient’s group, ensuring that any existing difference in mean between groups was kept. Explicitly, let *x_m,g,i,c_* be the observed expression, normalized and log-transformed, of cell marker *m* for cell *c* of patient *i*, belonging to group *g*; let *N_g_* be the total number of cells in group *g* and *N_g,i_* the number of cells from patient *i*, with 
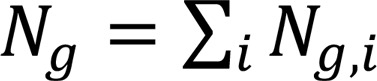
.

The mean expression of the marker for patient *i* is


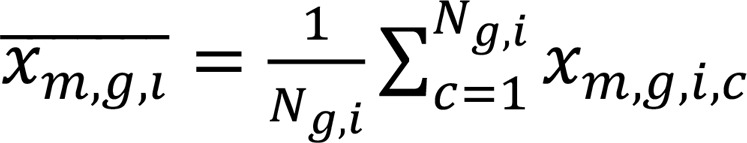
;

the mean expression for the whole group *g* is


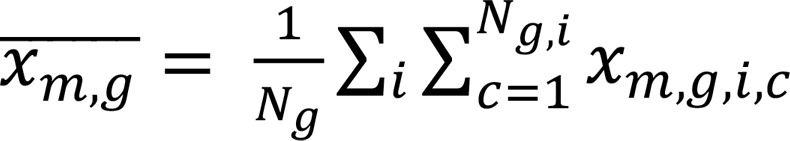
.

Then, the harmonized version is


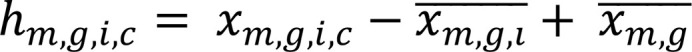
.

With this correction, the mean for all cells of a patient of a given group was equal to the group mean, hence patient effects were canceled, but between-group differences (that is, differences between 
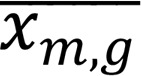
 values) were kept. Similarly, batch effect was removed so that mean for marker *m* was the same for all batches. In both cases, the correction was done using a linear model approach, by subtracting the difference to remove the values of all concerned droplets. Clustering was done next with Seurat, using the community detection algorithm (Louvain algorithm, resolution: 0.4) on the *k*-nearest neighbors graphs on the UMAP components. The UMAP was done on the first 20 PCA components. The PCA was performed on the 30 more variable surface markers, as obtained with the default Seurat parameters (FindVariableFeatures function), after centering and reducing.

Cluster abundance in a patient was defined as the number of cells in a cluster divided by the total number of cells (that is, as the proportion of the cells of the patient that are in the cluster). Changes in cluster abundance among patients were tested using a compositional data approach, as described in ref. 65, using the SARP.compo package for R (65). Briefly, since the sum of cluster abundances is, by definition, 1 in each patient, any change in a cluster abundance will translate into changes in opposite directions to all other cluster abundances, leading to compositional data. Hence, individual changes in a cluster abundance are not interpretable. However, changes in the ratio of the abundances of 2 clusters are interpretable. To interpret changes in all possible ratios, a graph is built: each node is a cluster, and 2 nodes are linked by an edge if their ratio is the same between R1 and R2 patients. If the final graph is disjoint (i.e., there are nodes for which no path exists to another node using the remaining edges), then corresponding cluster abundances change between R1 and R2 patients. An edge is cut if the corresponding test of the ratio between R1 and R2 is significant at a predefined threshold (*P* < threshold; 2-tailed *t* test on log-transformed abundance ratios). The threshold was determined by 10,000 simulations, to ensure a type I error rate (wrongly obtaining a disjoint graph) of α = 5% (considering multiple comparisons and association between tests). For 41 nodes and the CITE-Seq R1 and R2 sample sizes, the threshold was set at 0.38, ensuring a conservative test (simulation results: 0.393, 95% confidence interval 0.38–0.41).

Cluster annotation was done using (a) a list of the more expressed surface markers and less expressed surface markers, and (b) box plots of individual cell surface marker expression.

#### CITE-Seq data analysis of transcriptome.

Transcriptome RNA analysis was done separately for each cluster defined in the previous step. To circumvent the cell misclassification noise, any patient group (D/R1/R2) accounting for less than 10% of the total number of cells in a cluster was excluded from the transcriptomic analysis. With this threshold, we assume that clusters describe better their predominant bone marrow cell populations, while assuring the presence of enough cells for the differential analysis (i.e., more than 100).

To avoid systematic zeros in the transcriptomic analysis, genes for which transcripts were not detected in any cell of the given cluster were excluded; the list of these genes, totally absent in the cluster but present in at least another cluster, was kept and used to consolidate the cluster identification. Remaining genes were split into subsets of genes expressed in 1, 2, or 3 patient groups. For genes expressed in only 1 group, or if there was only 1 group in the cluster, the transcripts were characterized as average gene expressions. Otherwise, in addition to descriptive statistics, a differential analysis was done on pairwise comparisons of the patient groups in the cluster: controls against recipients 1 (D/R1), controls against recipients 2 (D/R2), and recipients 1 against recipients 2 (R1/R2). Following the systematic review of scRNA-Seq pipelines by Vieth et al. (66) and also as previously reported by Soneson and Robinson (67), we conducted the differential analysis with the limma-trend method (68, 69) associated with the scran normalization (70). Since both scran and limma procedures use raw counts data, the harmonization step described above for surface markers could not be done. Instead, since limma method is based on mixed-effects linear modeling, in addition to fixed group effect, batch effect was included as a fixed effect in the model, and patient effect as a random effect. The no-intercept model was used, and pairwise comparisons were obtained from the contrast analysis.

For the differential analyses, *P* values and the corresponding log fold changes are illustrated in volcano plots. Different multiplicity correction methods were used (Holm or Bonferroni-Hochberg; see figure legends) with a significance level set to α = 5%. Only a selection of genes is labeled, for better readability. In clusters where the 3 groups of patients were present, row count averages on the log_2_ scale were transformed into barycentric coordinates and represented as Triwise plots (R package Triwise, ref. 71). Reduced color saturation indicates non-significant expressions from pairwise patient group comparisons.

Genes tested with limma-trend in a given subset of transcripts were used as keys to select records from the annotation database org.Hs.eg.db (R package, ref. 72). Genes identified in this database were sorted in decreasing order according to corresponding limma-trend log fold changes and underwent a universal gene set enrichment analysis (GSEA) (clusterProfiler R package, ref. 73). We tested the overlap of the annotated genes with those from the hallmark gene sets (GSEA collection H) and immunologic signature gene sets (GSEA collection C7), retrieved from the Molecular Signatures Database (msigdbr R package, ref. 74). To control the type I error, the Benjamini-Hochberg adjusted *P* values were used with a cutoff set to 5%. The dot plots were generated with enrichplot (Yu et al., 2023, enrichplot R package, https://yulab-smu.top/biomedical-knowledge-mining-book/), to show the enrichment results ordered by gene ratios. Up to 20 most significant pathways are depicted per cluster. All detected pathways of all clusters are represented as a dot plot that features both log-transformed *P* values and enrichment scores.

### Statistics

Comparisons between study controls and recipient groups were performed using the Mann-Whitney test. The Kruskal-Wallis test (Dunn’s multiple comparisons) was used to compare controls, R1 recipients, and R2 recipients among patients within the same cell population. Comparisons of patients’ characteristics were performed by Fisher’s exact or χ^2^ tests. *P* values of less than 0.05 were considered significant (*P < 0.05, **P < 0.01, ***P < 0.001, ****P < 0.0001). Statistical analyses were performed using R or Prism.

### Study approval

Written informed consent was obtained from all participants in accordance with the Declaration of Helsinki. This study was declared to the Commission National Informatique et Liberté (CNIL) and was approved by the local ethics committee and Institutional Review Board (Comité de Protection des Personnes [CPP] Sud Est II, Paris, France; RCB number 2020- A01930-39).

### Data and materials availability

Data for mass cytometry are available in the FlowRepository (http://flowrepository.org; accession number FR-FCM-Z7Y7). Data for single-cell RNA-Seq are deposited in the NCBI’s Gene Expression Omnibus (GEO) database (GEO GSE249722). Scripts for analyses are available in GitLab (http://antiphishing.aphp.fr/v4?f=aDkzbzA4cUNkeDdOOUQzWiyYATI3qgX6dqBbRuEGThjFdElm3uIG9IJhtwp3ni7xWlZ4f-spzaWcCmkYooiwTA&i=clVIbUJReENOYndSaVlWWm_TWE6NT-7Tm6_rf5YiDcM&k=vhtD&r=cEVEckRRVXBqNE85QUxNY4p40P_6epCVpIhh6PRfdYhC_pNFKs-5WH8a1mvAQjss&s=3de6a92254c4ea6e784850427cbdbd21b2e71e0b6666d630cd34bbf25aee6885&u=https%3A%2F%2Fgitlab.com%2Fur-7537-biostm.saint-louis%2FB-REC).

Supporting data values associated with the main article and supplemental material, including values for all data points shown in graphs and values behind any reported means, are included in the Supporting Data Values file.

## Author contributions

GS conceptualized the study. E Curis, DM, DG, and GS designed the study methodology. AC performed mass cytometry experiments. JB, DG, DM, NV, MC, PL, SM, and E Clappier performed experiments. DM, MR, AX, FSDF, SCZ, RPDL, and GS recruited patients. JB and GS performed CyTOF data analysis. JB, DG, E Curis, and GS performed CITE-Seq analysis. GS acquired funding. GS and E Curis supervised the study. GS, JB, DB, and E Curis wrote the original draft of the manuscript. All authors contributed to the writing, reviewing, and editing of the manuscript.

## Supplementary Material

Supplemental data

ICMJE disclosure forms

Supporting data values

## Figures and Tables

**Table 1:**
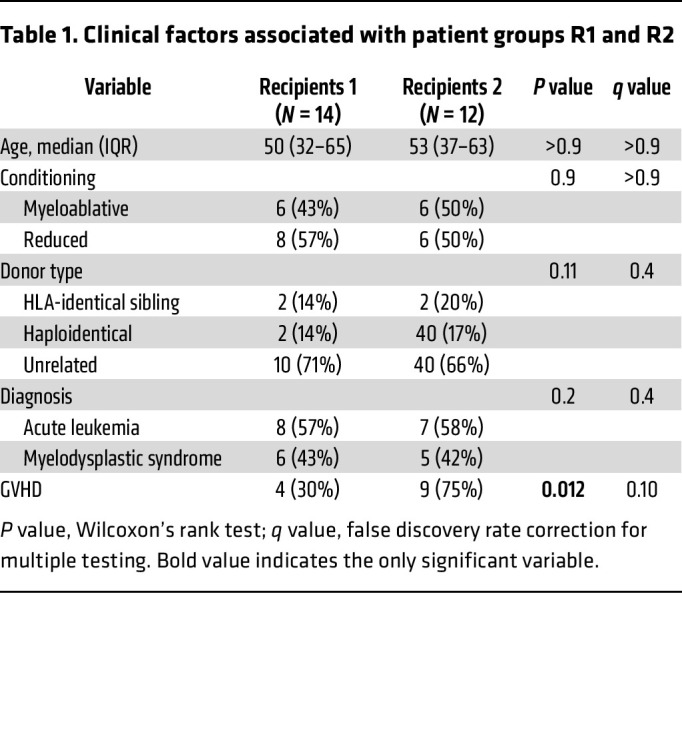
Clinical factors associated with patient groups R1 and R2

## Version 1 08/29/2024

In-Press Preview

## Version 2 10/15/2024

Electronic publication

## References

[1] Socie G, Michonneau D (2022). Milestones in acute GVHD pathophysiology. Front Immunol.

[2] Busch K, Rodewald HR (2016). Unperturbed vs. post-transplantation hematopoiesis: both in vivo but different. Curr Opin Hematol.

[4] Triana S (2021). Single-cell proteo-genomic reference maps of the hematopoietic system enable the purification and massive profiling of precisely defined cell states. Nat Immunol.

[5] Velten L (2017). Human haematopoietic stem cell lineage commitment is a continuous process. Nat Cell Biol.

[6] Weinreb C (2020). Lineage tracing on transcriptional landscapes links state to fate during differentiation. Science.

[7] Höfer T, Rodewald HR (2018). Differentiation-based model of hematopoietic stem cell functions and lineage pathways. Blood.

[8] Biasco L (2016). In vivo tracking of human hematopoiesis reveals patterns of clonal dynamics during early and steady-state reconstitution phases. Cell Stem Cell.

[9] Scala S (2018). Dynamics of genetically engineered hematopoietic stem and progenitor cells after autologous transplantation in humans. Nat Med.

[10] Velardi E (2021). T cell regeneration after immunological injury. Nat Rev Immunol.

[11] Sarantopoulos S, Ritz J (2015). Aberrant B-cell homeostasis in chronic GVHD. Blood.

[12] Allen JL (2014). Increased BCR responsiveness in B cells from patients with chronic GVHD. Blood.

[13] Allen JL (2012). B cells from patients with chronic GVHD are activated and primed for survival via BAFF-mediated pathways. Blood.

[14] Kuzmina Z (2011). Significant differences in B-cell subpopulations characterize patients with chronic graft-versus-host disease-associated dysgammaglobulinemia. Blood.

[15] Greinix HT (2008). Elevated numbers of immature/transitional CD21-B lymphocytes and deficiency of memory CD27+ B cells identify patients with active chronic graft-versus-host disease. Biol Blood Marrow Transplant.

[16] Pulendran B, Davis MM (2020). The science and medicine of human immunology. Science.

[17] Poe JC (2023). Single-cell landscape analysis unravels molecular programming of the human B cell compartment in chronic GVHD. JCI Insight.

[18] Glauzy S (2014). Impact of acute and chronic graft-versus-host disease on human B-cell generation and replication. Blood.

[19] Corre E (2010). Long-term immune deficiency after allogeneic stem cell transplantation: B-cell deficiency is associated with late infections. Haematologica.

[20] Bendall SC (2014). Single-cell trajectory detection uncovers progression and regulatory coordination in human B cell development. Cell.

[21] Glass DR (2020). An integrated multi-omic single-cell atlas of human B cell identity. Immunity.

[22] Ikeda N (2023). The early neutrophil-committed progenitors aberrantly differentiate into immunoregulatory monocytes during emergency myelopoiesis. Cell Rep.

[23] Huo Y (2023). Single-cell dissection of human hematopoietic reconstitution after allogeneic hematopoietic stem cell transplantation. Sci Immunol.

[24] Aiuti A (2013). Lentiviral hematopoietic stem cell gene therapy in patients with Wiskott-Aldrich syndrome. Science.

[25] Six E (2020). Clonal tracking in gene therapy patients reveals a diversity of human hematopoietic differentiation programs. Blood.

[26] Montaldo E (2022). Cellular and transcriptional dynamics of human neutrophils at steady state and upon stress. Nat Immunol.

[27] Shono Y (2010). Bone marrow graft-versus-host disease: early destruction of hematopoietic niche after MHC-mismatched hematopoietic stem cell transplantation. Blood.

[28] Pierini A (2017). Foxp3^+^ regulatory T cells maintain the bone marrow microenvironment for B cell lymphopoiesis. Nat Commun.

[29] Fedoriw Y (2012). Bone marrow B cell precursor number after allogeneic stem cell transplantation and GVHD development. Biol Blood Marrow Transplant.

[30] Mensen A (2014). Bone marrow T-cell infiltration during acute GVHD is associated with delayed B-cell recovery and function after HSCT. Blood.

[31] Lakshmikanth T (2017). Mass cytometry and topological data analysis reveal immune parameters associated with complications after allogeneic stem cell transplantation. Cell Rep.

[32] Gournay V (2022). Immune landscape after allo-HSCT: TIGIT- and CD161-expressing CD4 T cells are associated with subsequent leukemia relapse. Blood.

[33] Nakamae H (2011). Cytopenias after day 28 in allogeneic hematopoietic cell transplantation: impact of recipient/donor factors, transplant conditions and myelotoxic drugs. Haematologica.

[34] Masouridi-Levrat S (2016). Immunological basis of bone marrow failure after allogeneic hematopoietic stem cell transplantation. Front Immunol.

[35] Larocca A (2006). Boost of CD34+-selected peripheral blood cells without further conditioning in patients with poor graft function following allogeneic stem cell transplantation. Haematologica.

[36] Prabahran A (2021). Evaluation of risk factors for and subsequent mortality from poor graft function (PGF) post allogeneic stem cell transplantation. Leuk Lymphoma.

[37] Prabahran A (2022). Non-relapse cytopenias following allogeneic stem cell transplantation, a case based review. Bone Marrow Transplant.

[38] Radtke S (2023). Stochastic fate decisions of HSCs after transplantation: early contribution, symmetric expansion, and pool formation. Blood.

[39] Camacho V (2020). Bone marrow Tregs mediate stromal cell function and support hematopoiesis via IL-10. JCI Insight.

[40] Xie X (2020). Single-cell transcriptome profiling reveals neutrophil heterogeneity in homeostasis and infection. Nat Immunol.

[41] Young NS (2018). Aplastic anemia. N Engl J Med.

[42] Singh P, Ali SA (2022). Multifunctional role of S100 protein family in the immune system: an update. Cells.

[43] Urbanus J (2023). DRAG in situ barcoding reveals an increased number of HSPCs contributing to myelopoiesis with age. Nat Commun.

[44] Lambert K (2022). Deep immune phenotyping reveals similarities between aging, Down syndrome, and autoimmunity. Sci Transl Med.

[45] Colom Díaz PA (2023). Hematopoietic stem cell aging and leukemia transformation. Blood.

[46] Munz CM (2023). Regeneration after blood loss and acute inflammation proceeds without contribution of primitive HSCs. Blood.

[47] Caiado F (2021). Inflammation as a regulator of hematopoietic stem cell function in disease, aging, and clonal selection. J Exp Med.

[48] Chavakis T (2022). Inflammatory modulation of hematopoiesis: linking trained immunity and clonal hematopoiesis with chronic disorders. Annu Rev Physiol.

[49] Collins A (2021). Inflammatory signaling regulates hematopoietic stem and progenitor cell development and homeostasis. J Exp Med.

[50] Fanti AK (2023). Flt3- and Tie2-Cre tracing identifies regeneration in sepsis from multipotent progenitors but not hematopoietic stem cells. Cell Stem Cell.

[51] Yamashita M, Passegué E (2019). TNF-α coordinates hematopoietic stem cell survival and myeloid regeneration. Cell Stem Cell.

[52] Zoumbos NC (1985). Interferon is a mediator of hematopoietic suppression in aplastic anemia in vitro and possibly in vivo. Proc Natl Acad Sci U S A.

[53] Selleri C (1995). Interferon-gamma and tumor necrosis factor-alpha suppress both early and late stages of hematopoiesis and induce programmed cell death. J Cell Physiol.

[54] Alvarado LJ (2019). Eltrombopag maintains human hematopoietic stem and progenitor cells under inflammatory conditions mediated by IFN-γ. Blood.

[55] Strati P (2023). Prolonged cytopenia following CD19 CAR T cell therapy is linked with bone marrow infiltration of clonally expanded IFNγ-expressing CD8 T cells. Cell Rep Med.

[56] Patel BK (2023). Blood group A enhances SARS-CoV-2 infection. Blood.

[57] Martin PJ (1995). Influence of alloreactive T cells on initial hematopoietic reconstitution after marrow transplantation. Exp Hematol.

[58] Martin PJ (1990). The role of donor lymphoid cells in allogeneic marrow engraftment. Bone Marrow Transplant.

[59] Maraninchi D (1987). Impact of T-cell depletion on outcome of allogeneic bone-marrow transplantation for standard-risk leukaemias. Lancet.

[60] Champlin RE (2000). T-cell depletion of bone marrow transplants for leukemia from donors other than HLA-identical siblings: advantage of T-cell antibodies with narrow specificities. Blood.

[61] Soiffer RJ (2011). Impact of immune modulation with anti-T-cell antibodies on the outcome of reduced-intensity allogeneic hematopoietic stem cell transplantation for hematologic malignancies. Blood.

[62] Baker MB (1997). Graft-versus-host-disease-associated lymphoid hypoplasia and B cell dysfunction is dependent upon donor T cell-mediated Fas-ligand function, but not perforin function. Proc Natl Acad Sci U S A.

[63] van Dongen JJM (2003). Design and standardization of PCR primers and protocols for detection of clonal immunoglobulin and T-cell receptor gene recombinations in suspect lymphoproliferations: report of the BIOMED-2 Concerted Action BMH4-CT98-3936. Leukemia.

[64] Duez M (2016). Vidjil: a web platform for analysis of high-throughput repertoire sequencing. PLoS One.

[65] Curis E (2019). Determination of sets of covariating gene expression using graph analysis on pairwise expression ratios. Bioinformatics.

[66] Vieth B (2019). A systematic evaluation of single cell RNA-seq analysis pipelines. Nat Commun.

[67] Soneson C, Robinson MD (2018). Bias, robustness and scalability in single-cell differential expression analysis. Nat Methods.

[68] Law CW (2014). voom: precision weights unlock linear model analysis tools for RNA-seq read counts. Genome Biol.

[69] Ritchie ME (2015). limma powers differential expression analyses for RNA-sequencing and microarray studies. Nucleic Acids Res.

[70] McLaren W (2016). The ensembl variant effect predictor. Genome Biol.

[71] van de Laar L (2016). Yolk sac macrophages, fetal liver, and adult monocytes can colonize an empty niche and develop into functional tissue-resident macrophages. Immunity.

[72] https://bioconductor.org/packages/release/data/annotation/html/org.Hs.eg.db.html.

[73] Wu T (2021). clusterProfiler 4.0: a universal enrichment tool for interpreting omics data. Innovation (Camb).

[74] https://cran.r-project.org/web/packages/msigdbr/msigdbr.pdf.

