# Supplementary material for: Deciphering bone marrow engraftment after allogeneic stem cell transplantation in humans using single-cell analyses
Source: J Clin Invest. 2024 Aug 29;134(20):e180331. doi: 10.1172/JCI180331 (PMC11473149; doi:10.1172/JCI180331)
Supplement: Supplemental data [file jci-134-180331-s037.pdf]

Deciphering engraftment of bone marrow human single cells after allogeneic stem cell transplantation

Supplementary Figures & Tables

As appear in the main text

Table S1: Patient-, disease- and transplant-characteristics; Overall cohort

Parameter	N = 42
**Age (years; range)**	52 (24-69)
**Gender (Male/Female; N (%))**	26 (62%) /16 (38%)
**Conditioning regimen (RIC/MAC; N (%))**	30 (71%) /12 (29%)
**Control type**	
HLA-identical sibling (N (%))	15(36%)
Haploidentical (N (%))	12 (28%)
Matched Unrelated (N (%))	15 (36%)
**Diagnosis (N (%))**	
Myelodysplastic syndrome	18 (43%)
Acute myelogenous leukemia	15 (36%)
Acute bi phenotypic leukemia	5 (12%)
Other	4 (9%)

RIC: reduced intensity conditioning; MAC: myeloablative conditioning

Table S2: Population of patients with bone marrow analyses; Patient-, disease-, and transplant-characteristics.

	Global (N=26)	Recipient cluster 1 (N=14)	Recipient cluster 2 (N=12)
**Gender**			
**Age (years; range)**	51 (29-68)	50 (29-65)	51 (27-68)
**Diagnosis**			
**Acute myeloid leukemia (N, %)**	11/26 (42%)	6/14 (42.8%)	5/12 (41.7%)
Myelodysplastic syndrome (N, %)	12/26 (46%)	7/14 (50%)	5/12 (41.7%)
Acute bi phenotypic leukemia (N, %)	3 (12%)	1/14 (7.2%)	2/12 (16.7%)
**Control type (N; %)**			
HLA identical sibling	4 (15%)	2 (14%)	2 (16%)
Haploidentical	7 (27%)	2 (14%)	5 (42%)
Matched unrelated	15 (58%)	10 (72%)	5 (42%)
**Conditioning regimen***			
Reduced intensity.	14 (54%)	8 (57%)	6 (50%)
Myeloablative	12 (46%)	6 (43%)	6 (50%)
**GVHD prophylaxis**			
**CSA / MMF**	12 (46%)	9 (64%)	3 (25%)
**CSA / MTX**	6 (23%)	3 (21%)	3 (25%)
PTCy / CSA /MMF	8 (31%)	2 (15%)	6 (50%)
**Acute GVHD Grade** (Before sampling)			
0	14	11	3
I-IV	13 (50%)	**4 (28%)**	**9 (75%)**
≥ II	11 (42%)	**4 (28%)**	**7 (58%)**
**Immunosuppressive drugs at sampling**			
CSA	16	12	4
CSA + prednisone	10/26 (38.5%)	**2/14 (14%)**	**8/12 (67%)**
**CMV serological status**			
D-/R+	3 (11.5%)	2 (14.5%)	1 (8%)
D-/R-	8 (31%)	4 (28.5%)	4 (34%)
D+/R-	4 (15%)	4 (28.5%)	0
D+R+	11 (42.5%)	4 (28.5%	7 (58%)
**Alive /Dead**	21/5	14/3	12/2
**Cause of death**		Relapse, COVID (2)	GVHD, relapse

Table S3: Bone marrow aspiration cytology

	All recipients N = 26	Recipient Cluster 1 N = 14	Recipient Cluster 2 N = 12
**Cellularity, n(%)**			
Rich	3 (12)	3 (21)	0 (0)
Normal	13 (50)	5 (36)	8 (57)
Average	7 (27)	4 (29)	3 (25)
Poor	4 (15)	2 (14)	2 (17)
**Granulocytic Lineage**	**52**	**50**	**54**
**(expected range;50-75%)**	0.83	0.84	0.82
Myeloblast	5.1	4.1	6.3
Promyelocyte	10.0	8.8	11.4
Myelocyte	12.1	11.3	13.0
Metamyelocyte	21.4	21.9	20.9
Neutrophil			
**Erythroblasts (expected range;10-30%)**	**33.3**	**30.8**	**36.2**
**Lymphocytes* (expected range;5-15%)**	**10.3**	**13.8**	**6.2**
**Monocytes (expected range;0-3%)**	**3.2**	**3.3**	**3**
**Megakaryocytes (cellularity)**			
Normal	12	8	4
Average	8	4	4
Poor	6	2	4

* 4 patients had detectable hematogones (3 in cluster 1, 1 in cluster 2)

Table S4A: Panel of antibodies used for mass cytometry analyses of bone marrow cell population (according to (*19*))

Metal	Target	Clone	Reference	Staining temp.	Dilution (µl/100µl)	Panel BMMNC	Panel PBMC	Inhouse labeling*
148Nd	CD34	581	3148001B	4°C	1	■	■	No
174Yb	CD38	HIT2	303535	4°C	0,5	■	■	Yes
172Yb	IgM	MHM-88	3172004B	4°C	0,5	■	■	No
145Nd	CD45RB	MEM-55	3145009B	4°C	0,5	■	■	No
153Eu	CD62L (L-selectin)	DREG-56	3153004B	4°C	0,5	■	■	No
144Nd	CD15 (SSEA-1)	W6D3	3144019B	4°C	1	■	■	No
176Yb	CD123 (IL-3R)	6H6	306027	4°C	0,25	■	■	Yes
173Yb	CD117 (c.kit)	104D2	313223	4°C	0,5	■		Yes
175Lu	CD71	OKT-9	3175011B	4°C	0,5	■		No
89Y	CD45	HI30	3089003B	4°C	0,5	■	■	No
143Nd	CD5	UCHT2	3143007B	4°C	0,5	■	■	No
147Sm	CD20	2H7	3147001B	4°C	0,5	■	■	No
169Tm	CD24	ML5	3169004B	4°C	1	■	■	No
167Er	CD27	L128	3167006B	4°C	0,5	■	■	No
160Gd	Ig kappa (light chain)	MHK-49	3160005B	4°C	0,5	■	■	No
151Eu	Ig lambda (light chain)	MHL-38	3151004B	4°C	0,75	■	■	No
146Nd	IgD	IA6-2	3146005B	4°C	0,25	■	■	No
170Er	CD45RA	HI100	3170010B	37°C	0,25	■	■	No
164Dy	TdT	E17-1519	3164015B	4°C	0,5	■	■	No
165Ho	CD127 (IL-7Ra)	A019D5	3165008B	4°C	0,5	■	■	No
150Nd	CD43	84-3C1	3150006B	4°C	0,5	■	■	No
154Sm	Pax5	1H9	14-9918-82	4°C	0,5	■	■	No
161Dy	CD9	HI9a	312102	4°C	0,25	■	■	No
171Yb	RAG1	1D9	MA517163	4°C	1	■	■	No
155Gd	CD23	EBVCS-5	338502	4°C	0,5	■	■	Yes
163Dy	CD72	3F3	316202	4°C	0,25	■	■	Yes
152Sm	CD21	BL13	3152010B	4°C	0,5	■	■	No
159Tb	CD22	HIB22	3159005B	4°C	0,75	■	■	No
142Nd	CD40	5C3	3142010B	4°C	1	■	■	No
158Gd	CD179b	HSL11	3158016B	4°C	0,25	■		No
149Sm	CD179a	HSL96	3149012B	4°C	0,5	■		No
162Dy	Integrin b7	FIB504	3162026B	37°C	0,5	■	■	No
141Pr	CD79B	CB3-1	14-0793-82	4°C	0,5	■	■	No
168Er	CD138	DL-101	3168009B	4°C	0,5	■		No
168Er	CD73	AD2	3168015B	4°C	0,5		■	No
156Gd	CD10	HI10a	3156001B	4°C	0,5	■	■	No
166Er	CD7	M-T701	3166027B	4°C	0,25	■		No
209Bi	CD11b	ICRF44	3209003B	4°C	0,25	■		No
Cd106	CD19	HIB19	302247	4°C	0,5	■	■	Yes
Cd110	IgG	HP6017	409302	4°C	0,5	■	■	Yes
Cd113	HLA-DR	L243	307651	4°C	0,5	■	■	Yes
Cd116	CD79a	HM47	333502	4°C	0,5	■	■	Yes

Temp: Temperature. *In house labeling was performed with MaxPar X8 and MCP9 reagents kit following manufacturer instructions. At the end of the procedure, recovered labelled antibodies were diluted to 1 mg/mL

Table S4B: Phenotypic definitions of bone marrow cell populations (according to (*19*))

Progenitor cells	
**Cell population**	**Phenotype**
Hematopoietic stem cell (HSC)	CD34^{+}CD38^{-} CD45^{-} Lin^{-}
Lymphoid-primed multipotent progenitor (LMPP)	CD34^{+}CD38^{+}CD117^{+}CD127^{-}
Common lymphoid progenitor (CLP)	CD34^{+}CD38^{+}CD117^{-}
Common myeloid progenitor (CMP)	CD34^{+}CD38^{+}CD123^{+}CD45RA^{-}

B cells	
**Cell population**	**Phenotype**
Pre-pro-B	CD34^{+}CD38^{+}CD127^{+}CD10^{+}
Pro-B	CD34^{+}CD38^{+}CD10^{+}CD24^{+}CD19^{low}
Pre-B	CD34^{low}CD38^{+}CD10^{int}CD24^{+}CD19^{+}
Immature B	CD38^{+}CD10$^{int/low}$CD24^{+}CD9^{+}CD19^{+}CD20^{+}IgM^{+}CD79b^{+}IgK$^{+/-}$ Igλ$^{+/-}$
Transitional B	CD38^{+}CD24^{+}CD19^{+}CD20^{+}IgM^{+}CD22^{+}CD40^{+}CD79b^{+}IgD$^{+/-}$ CD21^{+}IgK$^{+/-}$Igλ$^{+/-}$
Naive B	CD38^{-}CD24^{+}CD19^{+}CD20^{+}IgM^{+}CD22^{+}CD40^{+}CD79b^{+} IgD^{+}CD21^{+}CD27^{-}IgK$^{+/-}$Igλ$^{+/-}$
Plasma cells	CD38^{+}CD19^{low}CD138^{+}CD27^{+}

T cells	
**Cell population**	**Phenotype**
TEM cells (effector memory)	CD45^{+}CD5^{+}CD45RA^{-}CD27^{-}
TEMRA cells	CD45^{+}CD5^{+}CD45RA^{+}CD27^{+}
Naive T cells	CD45^{+}CD5^{+}CD45RB^{+}CD45RA^{+}CD127^{+}CD27^{+}
T-cell Precursors	CD45^{+}CD38^{+}CD7^{+}CD5^{-}
T cells	CD45^{+}CD7^{+}CD5^{+}CD127^{+}CD27^{+}

Myeloid cells	
**Cell population**	**Phenotype**
Myeloid progenitors	CD45^{+}CD38^{low}CD10^{+}CD66b^{+}CD11b^{+}
Monocytes	CD45^{+}CD66b^{+}CD11b^{+}

Others cells	
**Cell population**	**Phenotype**
Granulocytes	CD38^{+}CD66b^{+}
Plasmacytoid DC	CD38^{+}CD62L^{+}CD123^{+}
Erythroid precursors	CD38^{low}CD71^{+}

Table S5A: Panel of antibodies used to study bone marrow T-cell populations.

Metal	Target	Clone	Reference	Staining temp.	Dilution (µl/100µl)
116CD	CD3	UCHT1	300443	4°C	1
11Cd	CD4	SK3	344625	4°C	0,13
141Pr	CD5	UCHT2	300627	4°C	0,25
113Cd	CD8	SK1	344727	4°C	0,25
112Cd	CD11b	ICRF44	301337	4°C	0,4
147Sm	CD11c	Bu15	3147008B	4°C	0,5
106Cd	CD14	M5E2	301843	4°C	0,75
144Nd	CD15	W6D3	3144019B	4°C	0,5
110Cd	CD19	HIB19	302247	4°C	0,5
169Tm	CD25	2A3	3169003B	4°C	0,5
158Gd	CD33	WM53	3158001B	4°C	0,25
149Sm	CD34	581	3149013B	4°C	1
160Gd	CD39	A1	3160004B	4°C	0,4
89Y	CD45	HI30	3089003B	4°C	0,5
176Yb	CD56	NCAM16.2	3176008B	4°C	0,25
168Er	CD73	AD2	3168015B	4°C	0,5
143Nd	CD117	104D2	3143001B	4°C	0,75
165Ho	CD127 (IL-7Ra)	A019D5	3165008B	4°C	1
175Lu	CD223/LAG-3	11C3C65	3175033B	4°C	1
164Dy	CD272 (BTLA)	MIH26	344502	4°C	0,5
174Yb	CD279 (PD-1)	EH12.2H7	3174020B	4°C	1
142Nd	TGIT	MBSA43	372702	4°C	1
154Sm	TIM-3	F38-2E2	3154010B	4°C	1,5
167Er	CD197 (CCR7)	G043H7	3167009A	37°C	0,5
155Gd	CD45RA	HI100	3155011B	37°C	0,5
173Yb	CD194 (CCR4)	L291H4	359402	37°C	0,3
162Dy	FoxP3	259D/C7	3162024A	4°C	1,25
171Yb	Granzyme B	GB11	3171002B	4°C	0,5

Table S5B: Phenotypic definitions of bone marrow T-cell populations in CyTOF analyses

Progenitor cells	
**Cell population**	**Phenotype**
Hematopoietic stem and progenitor cells (HSPC)	$CD34^{+}CD45^{low}$

T cells	
**Cell population**	**Phenotype**
CD4+ TEM cells	$CD45^{+}CD3^{+}CD5^{+}CD4^{+}CD45RA^{-}CD127^{+/-}CD197^{-}$
CD4+ TCM cells	$CD45^{+}CD3^{+}CD5^{+}CD4^{+}CD45RA^{-}CD127^{+/-}CD197^{+}$
CD4+ Treg cells	$CD45^{+}CD3^{+}CD5^{+}CD4^{+}CD45RA^{+/-}$ $CD127^{low}CD25^{+}$
CD4+ CCR4+ $T_{EM/CM}$ cells	$CD45^{+}CD3^{+}CD5^{+}CD4^{+}CD45RA^{+/-}CD127^{+/-}CD197^{+/-}CD194^{+}$
CD4+ Naive T cells	$CD45^{+}CD3^{+}CD5^{+}CD4^{+}CD45RA^{+}CD127^{+/-}CD197^{+}$
Innate-like $CD4^{+}$ T cells	$CD45^{+}CD3^{+}CD5^{+}CD4^{+}CD45RA^{-}CD127^{+/-}CD56^{+}$
DN T cells	$CD45^{+}CD3^{+}CD5^{+}CD4^{-}CD8^{-}CD127^{+/-}CD45RA^{+/-}$
CD8+ TEM cells	$CD45^{+}CD3^{+}CD5^{+}CD8^{+}CD45RA^{-}CD127^{+/-}CD197^{-}$
CD8+ TEM/CM cells	$CD45^{+}CD3^{+}CD5^{+}CD8^{+}CD45RA^{-}CD127^{+}$
CD8+ TEMRA cells	$CD45^{+}CD3^{+}CD5^{+}CD8^{+}CD45RA^{+}CD127^{+/-}CD197^{+/-}CD194^{+}$
CD8+ Naive T cells	$CD45^{+}CD3^{+}CD5^{+}CD8^{+}CD45RA^{+}CD127^{+/-}CD197^{+}$
Innate-like $CD8^{+}$ T cells	$CD45^{+}CD3^{+}CD5^{+}CD8^{+}CD45RA^{-}CD127^{+/-}CD56^{+}$

Natural killer cells	
**Cell population**	**Phenotype**
NK	$CD56^{+}$
NK 56^{bright}	$CD56^{bright/high}$

Myeloid cells	
**Cell population**	**Phenotype**
Precursors Myeloid	$CD45^{+}CD33^{+}CD11b^{-}CD14^{-}$
Monocytes	$CD45^{+}CD33^{+}CD11b^{+}CD14^{low}$

Other cells	
**Cell population**	**Phenotype**
B cells	$CD45^{+}CD19^{+}$
Non-immune cells	$CD45^{-}$
T-mono complex	$CD45^{+}CD3^{+}CD5^{+}CD4^{+}CD8^{+}CD45RA^{-}CD127^{+/-}CD11b^{+}CD14^{+}$

Table S6A: TotalSeq™-C Human Universal antibody used in CITEseq experiments.

Description	Clone	Barcode	Name
**anti-human CD86**	IT2.2	GTCTTTGTCAGTGCA	CD86
**anti-human CD274 (B7-H1, PD-L1)**	29E.2A3	GTTGTCCGACAATAC	CD274
**anti-human CD270 (HVEM, TR2)**	122	TGATAGAAACAGACC	TNFRSF14
**anti-human CD155 (PVR)**	SKII.4	ATCACATCGTTGCCA	PVR
**anti-human CD112 (Nectin-2)**	TX31	AACCTTCCGTCTAAG	NECTIN2
**anti-human CD47**	CC2C6	GCATTCTGTCACCTA	CD47
**anti-human CD48**	BJ40	CTACGACGTAGAAGA	CD48
**anti-human CD40**	5C3	CTCAGATGGAGTATG	CD40
**anti-human CD154**	24-31	GCTAGATAGATGCAA	CD40LG
**anti-human CD52**	HI186	CTTTGTACGAGCAAA	CD52
**anti-human CD3**	UCHT1	CTCATTGTAACTCCT	CD3D
**anti-human CD8**	SK1	GCGCAACTTGATGAT	CD8A
**anti-human CD56**	5.1H11	TCCTTTCCTGATAGG	NCAM1
**anti-human CD19**	HIB19	CTGGGCAATTACTCG	CD19
**anti-human CD33**	P67.6	TAACTCAGGGCCTAT	CD33
**anti-human CD11c**	S-HCL-3	TACGCCTATAACTTG	ITGAX
**anti-human HLA-A,B,C**	W6/32	TATGCGAGGCTTATC	HLA-A
**anti-human CD45RA**	HI100	TCAATCCTTCCGCTT	PTPRC
**anti-human CD123**	6H6	CTTCACTCTGTCAGG	IL3RA
**anti-human CD7**	CD7-6B7	TGGATTCCCGGACTT	CD7
**anti-human CD105**	43A3	ATCGTCGAGAGCTAG	ENG
**anti-human/mouse CD49f**	GoH3	TTCCGAGGATGATCT	ITGA6
**anti-human CD194 (CCR4)**	L291H4	AGCTTACCTGCACGA	CCR4
**anti-human CD4**	RPA-T4	TGTTCCCGCTCAACT	CD4
**anti-mouse/human CD44**	IM7	TGGCTTCAGGTCCTA	CD44
**anti-human CD14**	M5E2	TCTCAGACCTCCGTA	CD14
**anti-human CD16**	3G8	AAGTTCACTCTTTGC	FCGR3A
**anti-human CD25**	BC96	TTTGTCCTGTACGCC	IL2RA
**anti-human CD45RO**	UCHL1	CTCCGAATCATGTTG	PTPRC
**anti-human CD279**	EH12.2H7	ACAGCGCCGTATTTA	PDCD1
**anti-human TIGIT (VSTM3)**	A15153G	TTGCTTACCGCCAGA	TIGIT
**Mouse IgG1, κ isotype Ctrl**	MOPC-21	GCCGGACGACATTAA	
**Mouse IgG2a, κ isotype Ctrl**	MOPC-173	CTCCTACCTAAACTG	
**Mouse IgG2b, κ isotype Ctrl**	MPC-11	ATATGTATCACGCGA	
**Rat IgG2b, κ Isotype Ctrl**	RTK4530	GATTCTTGACGACCT	
**anti-human CD20**	2H7	TTCTGGGTCCCTAGA	MS4A1
**anti-human CD335 (NKp46)**	9E2	ACAATTTGAACAGCG	NCR1
**anti-human CD31**	WM59	ACCTTTATGCCACGG	PECAM1
**anti-human CD146**	P1H12	CCTTGGATAACATCA	MCAM
**anti-human IgM**	MHM-88	TAGCGAGCCCGTATA	IGHM
**anti-human CD5**	UCHT2	CATTAACGGGATGCC	CD5
**anti-human CD183 (CXCR3)**	G025H7	GCGATGGTAGATTAT	CXCR3

**anti-human CD195 (CCR5)**	J418F1	CCAAAGTAAGAGCCA	CCR5
**anti-human CD32**	FUN-2	GCTTCCGAATTACCG	FCGR2A
**anti-human CD196 (CCR6)**	G034E3	GATCCCTTTGTCACT	CCR6
**anti-human CD185 (CXCR5)**	J252D4	AATTCAACCGTCGCC	CXCR5
**anti-human CD103 (Integrin αE)**	Ber-ACT8	GACCTCATTGTGAAT	ITGAE
**anti-human CD69**	FN50	GTCTCTTGGCTTAAA	CD69
**anti-human CD62L**	DREG-56	GTCCCTGCAACTTGA	SELL
**anti-human CD161**	HP-3G10	GTACGCAGTCCTTCT	KLRB1
**anti-human CD152 (CTLA-4)**	BNI3	ATGGTTCACGTAATC	CTLA4
**anti-human CD223 (LAG-3)**	11C3C65	CATTTGTCTGCCGGT	LAG3
**anti-human KLRG1 (MAFA)**	SA231A2	CTTATTTCCTGCCCT	KLRG1
**anti-human CD27**	O323	GCACTCCTGCATGTA	CD27
**anti-human CD107a (LAMP-1)**	H4A3	CAGCCCACTGCAATA	LAMP1
**anti-human CD95 (Fas)**	DX2	CCAGCTCATTAGAGC	FAS
**anti-human CD134 (OX40)**	Ber-ACT35 (ACT35)	AACCCACCGTTGTTA	TNFRSF4
**anti-human HLA-DR**	L243	AATAGCGAGCAAGTA	HLA-DRA
**anti-human CD1c**	L161	GAGCTACTTCACTCG	CD1C
**anti-human CD11b**	ICRF44	GACAAGTGATCTGCA	ITGAM
**anti-human CD64**	10,1	AAGTATGCCCTACGA	FCGR1A
**anti-human CD141 (Thrombomodulin)**	M80	GGATAACCGCGCTTT	THBD
**anti-human CD1d**	51,1	TCGAGTCGCTTATCA	CD1D
**anti-human CD314 (NKG2D)**	1D11	CGTGTTTGTTCCTCA	KLRK1
**anti-human CD35**	E11	ACTTCCGTCGATCTT	CR1
**anti-human CD57 Recombinant**	QA17A04	AACTCCCTATGGAGG	B3GAT1
**anti-human CD272 (BTLA)**	MIH26	GTTATTGGACTAAGG	BTLA
**anti-human/mouse/rat CD278 (ICOS)**	C398.4A	CGCGCACCCATTAAA	ICOS
**anti-human CD58 (LFA-3)**	TS2/9	GTTCCTATGGACGAC	CD58
**anti-human CD39**	A1	TTACCTGGTATCCGT	ENTPD1
**anti-human CX3CR1**	K0124E1	AGTATCGTCTCTGGG	CX3CR1
**anti-human CD24**	ML5	AGATTCCTTCGTGTT	CD24
**anti-human CD21**	Bu32	AACCTAGTAGTTCGG	CR2
**anti-human CD11a**	TS2/4	TATATCCTTGTGAGC	ITGAL
**anti-human CD79b (Igβ)**	CB3-1	ATTCTTCAACCGAAG	CD79B
**anti-human CD244 (2B4)**	C1.7	TCGCTTGGATGGTAG	CD244
**anti-human CD169 (Sialo adhesin, Siglec-1)**	7-239	TACTCAGCGTGTTTG	SIGLEC1
**anti-human/mouse integrin β7**	FIB504	TCCTTGGATGTACCG	ITGB7
**anti-human CD268 (BAFF-R)**	11C1	CGAAGTCGATCCGTA	TNFRSF13C
**anti-human CD42b**	HIP1	TCCTAGTACCGAAGT	GP1BB
**anti-human CD54**	HA58	CTGATAGACTTGAGT	ICAM1
**anti-human CD62P (P-Selectin)**	AK4	CCTTCCGTATCCCTT	SELP
**anti-human CD119 (IFN-γ R α chain)**	GIR-208	TGTGTATTCCCTTGT	IFNGR1

**anti-human TCR α/β**	IP26	CGTAACGTAGAGCGA	
**Rat IgG1, κ isotype Ctrl**	RTK2071	ATCAGATGCCCTCAT	
**Rat IgG2a, κ Isotype Ctrl**	RTK2758	AAGTCAGGTTCGTTT	
**Armenian Hamster IgG Isotype Ctrl**	HTK888	CCTGTCATTAAGACT	
**anti-human CD122 (IL-2Rβ)**	TU27	TCATTTCCTCCGATT	IL2RB
**anti-human CD267 (TACI)**	1A1	AGTGATGGAGCGAAC	TNFRSF13B
**anti-human FcεRIα**	AER-37 (CRA-1)	CTCGTTTCCGTATCG	FCER1A
**anti-human CD41**	HIP8	ACGTTGTGGCCTTGT	ITGA2B
**anti-human CD137 (4-1BB)**	4B4-1	CAGTAAGTTCGGGAC	TNFRSF9
**anti-human CD163**	GHI/61	GCTTCTCCTTCCTTA	CD163
**anti-human CD83**	HB15e	CCACTCATTTCCGGT	CD83
**anti-human CD124 (IL-4Rα)**	G077F6	CCGTCCTGATAGATG	IL4R
**anti-human CD13**	WM15	TTTCAACGCCCTTTC	ANPEP
**anti-human CD2**	TS1/8	TACGATTTGTCAGGG	CD2
**anti-human CD226 (DNAM-1)**	11A8	TCTCAGTGTTTGTGG	CD226
**anti-human CD29**	TS2/16	GTATTCCCTCAGTCA	ITGB1
**anti-human CD303 (BDCA-2)**	201A	GAGATGTCCGAATTT	CLEC4C
**anti-human CD49b**	P1E6-C5	GCTTTCTTCAGTATG	ITGA2
**anti-human CD81 (TAPA-1)**	5A6	GTATCCTTCCTTGGC	CD81
**anti-human IgD**	IA6-2	CAGTCTCCGTAGAGT	IGHD
**anti-human CD18**	TS1/18	TATTGGGACACTTCT	ITGB2
**anti-human CD28**	CD28.2	TGAGAACGACCCTAA	CD28
**anti-human CD38**	HIT2	TGTACCCGCTTGTGA	CD38
**anti-human CD127 (IL-7Rα)**	A019D5	GTGTGTTGTCCTATG	IL7R
**anti-human CD45**	HI30	TGCAATTACCCGGAT	PTPRC
**anti-human CD22**	S-HCL-1	GGGTTGTTGTCTTTG	CD22
**anti-human CD71**	CY1G4	CCGTGTTCCTCATTA	TFRC
**anti-human CD26**	BA5b	GGTGGCTAGATAATG	DPP4
**anti-human CD36**	5-271	TTCTTTGCCTTGCCA	CD36
**anti-human CD158 (KIR2DL1/S1/S3/S5)**	HP-MA4	TATCAACCAACGCTT	KIR2DL1
**anti-human CD49a**	TS2/7	ACTGATGGACTCAGA	ITGA1
**anti-human CD49d**	9F10	CCATTCAACTTCCGG	ITGA4
**anti-human CD73 (Ecto-5'-nucleotidase)**	AD2	CAGTTCCTCAGTTCG	NT5E
**anti-human TCR Vα7.2**	3C10	TACGAGCAGTATTCA	
**anti-human TCR Vδ2**	B6	TCAGTCAGATGGTAT	
**anti-human LOX-1**	15C4	ACCCTTTACCGAATA	OLR1
**anti-human CD158b (KIR2DL2/L3, NKAT2)**	DX27	GACCCGTAGTTTGAT	KIR2DL3
**anti-human CD158e1 (KIR3DL1, NKB1)**	DX9	GGACGCTTTCCTTGA	KIR3DL1
**anti-human CD319 (CRACC)**	162,1	AGTATGCCATGTCTT	SLAMF7
**anti-human CD99**	3B2/TA8	ACCCGTCCCTAAGAA	CD99
**anti-human CLEC12A**	50C1	CATTAGAGTCTGCCA	CLEC12A

**anti-human CD352 (NTB-A)**	NT-7	AGTTTCCACTCAGGC	SLAMF6
**anti-human CD94**	DX22	CTTTCCGGTCCTACA	KLRD1
**anti-human Ig light chain κ**	MHK-49	AGCTCAGCCAGTATG	IGKC
**anti-human CD85j (ILT2)**	GHI/75	CCTTGTGAGGCTATG	LILRB1
**anti-human CD23**	EBVCS-5	TCTGTATAACCGTCT	FCER2
**anti-human Ig light chain λ**	MHL-38	CAGCCAGTAAGTCAC	
**anti-human CD328 (Siglec-7)**	6-434	CTTAGCATTTCACTG	SIGLEC7
**anti-human GPR56**	CG4	GCCTAGTTTCCGTTT	ADGRG1
**anti-human HLA-E**	3D12	GAGTCGAGAAATCAT	HLA-E
**anti-human CD82**	ASL-24	TCCCACTTCCGCTTT	CD82
**anti-human CD101 (BB27)**	BB27	CTACTTCCCTGTCAA	CD101
**anti-human CD88 (C5aR)**	S5/1	GCCGCATGAGAAACA	C5AR1
**anti-human CD224**	KF29	CTGATGAGATGTCAG	GGT1

TotalSeq™-C Human Universal Cocktail, V1.0; BioLegend; cat# 399905

Table S6B: Antibodies added to the TotalSeq™-C Human Universal Cocktail for Cite-seq analyses.

Description	Clone	Barcode	Gene Name	Reference
**Anti-human CD9**	HI9a	GAGTCACCAATCTGC	5H9	312121
**Anti-human CD10**	HI10a	CAGCCATTCATTAGG		312233
**Anti-human CD117 (c-kit)**	104D2	AGACTAATAGCTGAC		313243
**Anti-human CD135 (Flt-3/Flk-2)**	BV10A4H2	CAGTAGATGGAGCAT		313323
**Anti-human CD34**	581	GCAGAAATCTCCCTT		343541
**Anti-human CD197**	G043H7	AGTTCAGTCAACCGA		353251
**Anti-human CD138**	MI15	ACTCTTTCGTTTACG		356539

Table S6C: Phenotypic definitions used to define cell populations in CITEseq experiments.

Progenitors cells	
**Cell population**	**Phenotype**
Lymphoid-primed multipotent progenitor (LMPP)	CD34^{+}CD38^{+}CD117^{+}CD127^{-}
Common lymphoid progenitor (CLP)	CD34^{+}CD38^{+}CD117^{-}

Myeloid cells	
**Cell population**	**Phenotype**
Common myeloid progenitor (CMP)	CD34^{+}CD38^{+}CD123^{+}CD45RA^{-}
Granulocyte and monocyte progenitor (GMP)	CD34^{+}CD38^{+}CD123^{+}CD45RA^{+}
Committed myeloid (MPP)	CD34^{low}CD38^{high}CD11b^{+}CD33^{+}CD16^{-}
Dendritic cells	CD38^{+}CD11c^{+}CD123^{low}CD11b^{+}
Classical monocyte	CD14^{++}CD16^{-}CD11b^{+}CD33^{+}
Intermediate monocyte	CD14$^{+/++}$CD16^{+}CD11b^{+}CD33^{+}
Non classical monocytes	CD14^{low}CD16^{++}CD11b^{+}CD33^{+}

B cells	
**Cell population**	**Phenotype**
Pre-pro-B	CD34^{+}CD38^{+}CD127^{+}CD10^{+}
Pro-B	CD34^{+}CD38^{+}CD10^{+}CD24^{+}CD19^{low}
Pre-B	CD34^{low}CD38^{+}CD10^{int}CD24^{+}CD19^{+}
Immature B	CD38^{+}CD10$^{int/low}$CD24^{+}CD9^{+}CD19^{+}CD20^{+} IgM^{+}CD79b^{+}
Transitional B	CD38^{+}CD24^{+}CD19^{+}CD20^{+}IgM^{+}CD22^{+}CD40^{+} CD79b^{+}IgD$^{+/-}$CD21^{+}
Naive B	CD38^{-}CD24^{+}CD19^{+}CD20^{+}IgM^{+}CD22^{+}CD40^{+} CD79b^{+}IgD^{+}CD21^{+}CD27^{-}
Plasma cells	CD38^{+}CD19^{low}CD138^{+}CD27^{+}

T cells	
**Cell population**	**Phenotype**
CD4^{+} T cells	CD45^{+}CD3^{+}CD5^{+}CD4^{+}CD45RA$^{+/-}$CD127$^{+/-}$CD197^{-}
CD4^{+} TSCM cells	CD45^{+}CD3^{+}CD5^{+}CD4^{+}CD45RO^{+}CD45RA^{+}CD95^{+}
Treg exhausted cells	CD45^{+}CD3^{+}CD5^{+}CD4^{+}CD45RA$^{+/-}$ CD127^{low}CD25^{+} CD73^{+}
CD279^{+}TIGIT^{+} T cells	CD45^{+}CD3^{+}CD5^{+}CD4$^{+/-}$CD8$^{+/-}$CD45RA^{-}CD127$^{+/-}$ CD197^{-}CD279^{+}TIGIT^{+}
CD4^{+} Naive T cells	CD45^{+}CD3^{+}CD5^{+}CD4^{+}CD45RA^{+}CD127$^{+/-}$CD197^{+}
NK T cells	CD45^{+}CD3^{+}CD5^{+}CD4$^{+/-}$CD8$^{+/-}$CD45RA$^{+/-}$CD127$^{+/-}$ CD56^{+}
CD8^{+} T cells	CD45^{+}CD3^{+}CD5^{+}CD8^{+}CD45RA^{-}CD127$^{+/-}$CD197^{-}

Natural killer cells	
**Cell population**	**Phenotype**
NK cells	CD56^{+}CD16^{+}
CD56^{bright} NK cells	CD56$^{bright/high}$CD16^{-}

Erythroblast cells	
CD34^{+} ERP early	CD34^{+}CD38^{+}CD36^{+}CD105^{+}CD10^{-}CD123^{-}CD45RA^{-}CD71^{+}
Megakaryocyte erythroid progenitor (MEP)	CD34^{low}CD38^{low}CD36^{+}CD105^{+}CD10^{-}CD123^{-}CD45RA^{-}CD71^{+}
Erythroblast (1)	CD34^{-}CD36^{+}CD105^{low}
Erythroblast (2)	CD34^{-}CD36^{int}CD105^{low}

Others cells	
**Cell population**	**Phenotype**
Mesenchymal stem cells (MSC)	CD34$^{-/low}$CD45^{-}CD105^{+}CD90^{+}CD73^{+}
Eosinophil	CD45^{+}CD11b^{+}CD46d^{+}CD14^{low} (based on expression of RNA)
CD45^{neg} cells	CD45^{neg}

Figure S1: Overall cohort (42 patients) ; samples repartition: In 16 patients only PBMC were studied. were studied (* Some patients had missing samples at 3 or 6 months: thus, numbers do not add to n=16). In 26 patients bone marrow cells were analyzed with 22/26 being also studied on PBMC

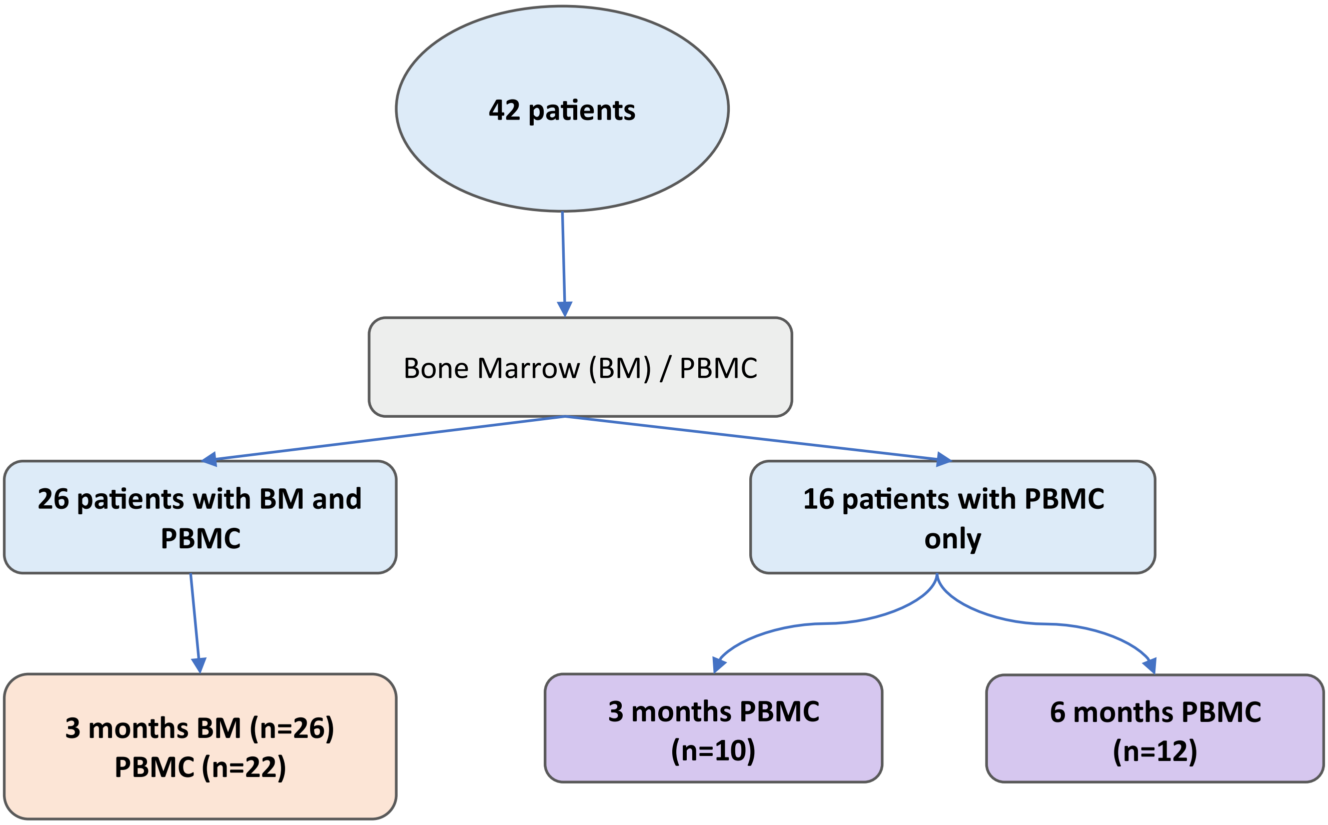

Figure S2: Peripheral Blood; parameters (A) and chimerism analyses (B)

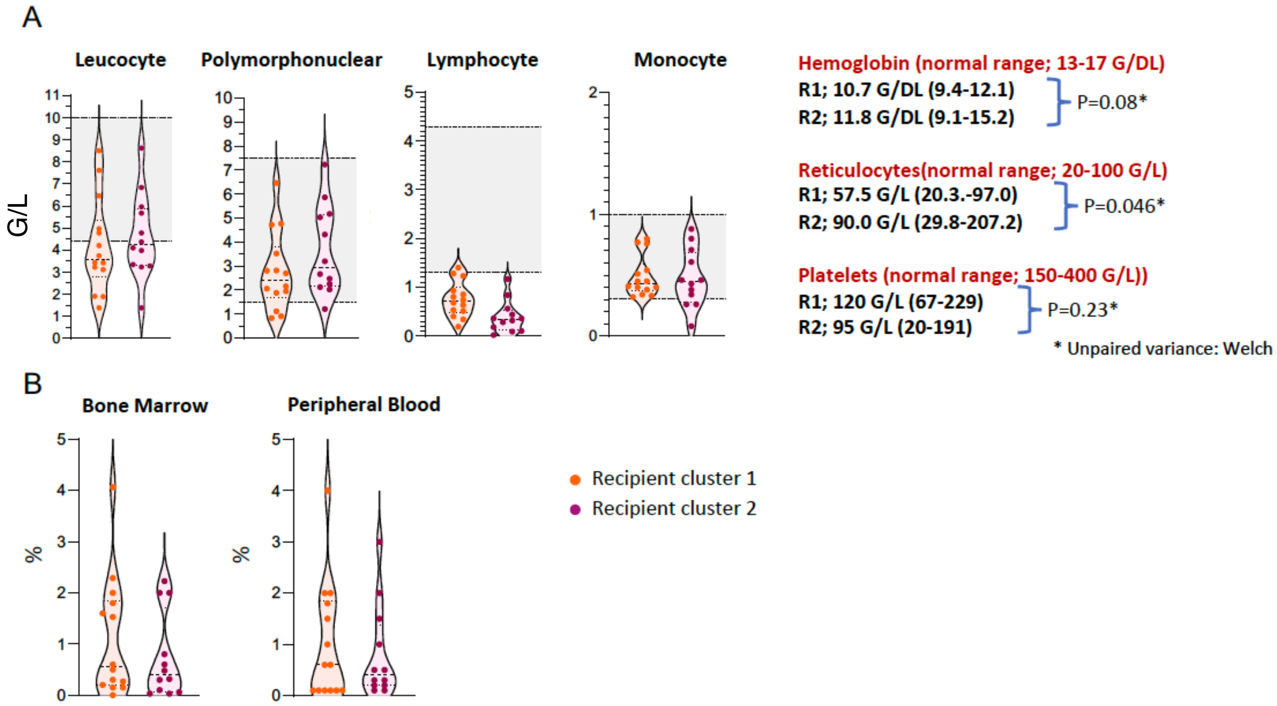

(A) Numeration of leucocyte from peripheral blood in recipient of cluster 1 (n=14) and recipient of cluster 2 (n=12). The normal numeration from controls is represented by gray square. **(B)** Chimerism analyses (as expressed by % of cells of recipient origin) from bone marrow and peripheral blood (using KMR kit, from GenDX ®). As detailed infra, these patients were segregated into two groups [Recipients 1 (R1) and recipients 2 (R2)], but except for reticulocyte counts R1 and R2 do not differ significantly for peripheral blood parameters.

Figure S3: Peripheral Blood mass cytometry analyses of B cell subsets

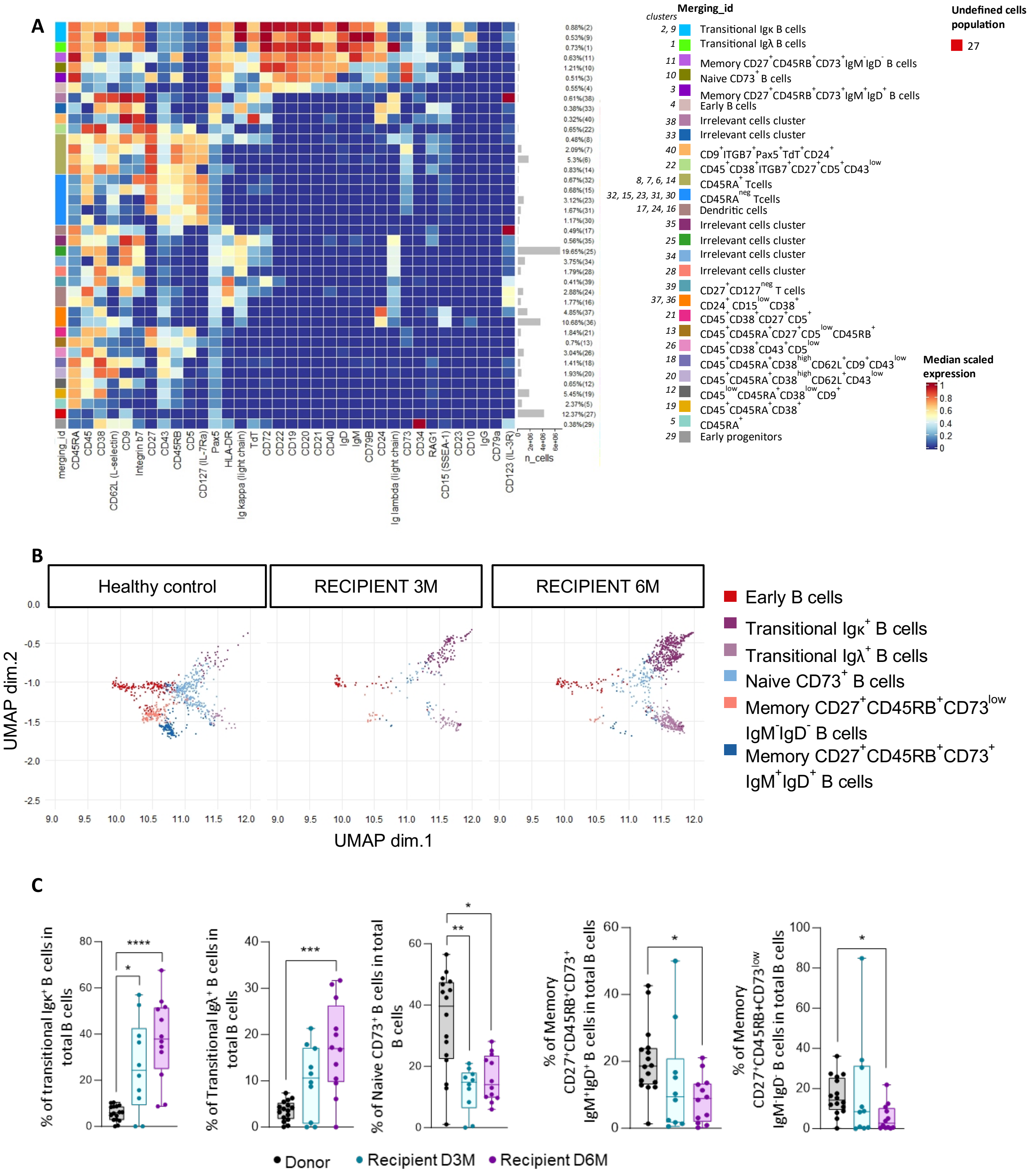

(A) Heatmap showing median expression intensities of each protein marker (columns), for each detected cluster (rows) using FlowSOM algorithm, with 40 indicated relevant metaclusters. **(B)** UMAP visualization of B cells populations from human CD15^{neg} Bone marrow mononuclear cells (BMMC) controls (n=16), recipient at 3 months post-transplantation (D3M) (n=10) and recipients at 6 months post-transplantation (D6M) (n=12) patients. B cells populations were defined by FlowSOM in figure S3A. Colors indicate clusters. **(C)** Quantification of percentage of B cells for all subsets from human CD15^{neg} BMMC heathy donors, recipient at 3 months and 6 months after transplantation. P-values correspond to Kruskal-Wallis test: *p <0.05; **p<0.01, ***p<0.001; ****p<0.0001. Donor refers to healthy controls.

Figure S4: Staining panel design of human bone marrow data set

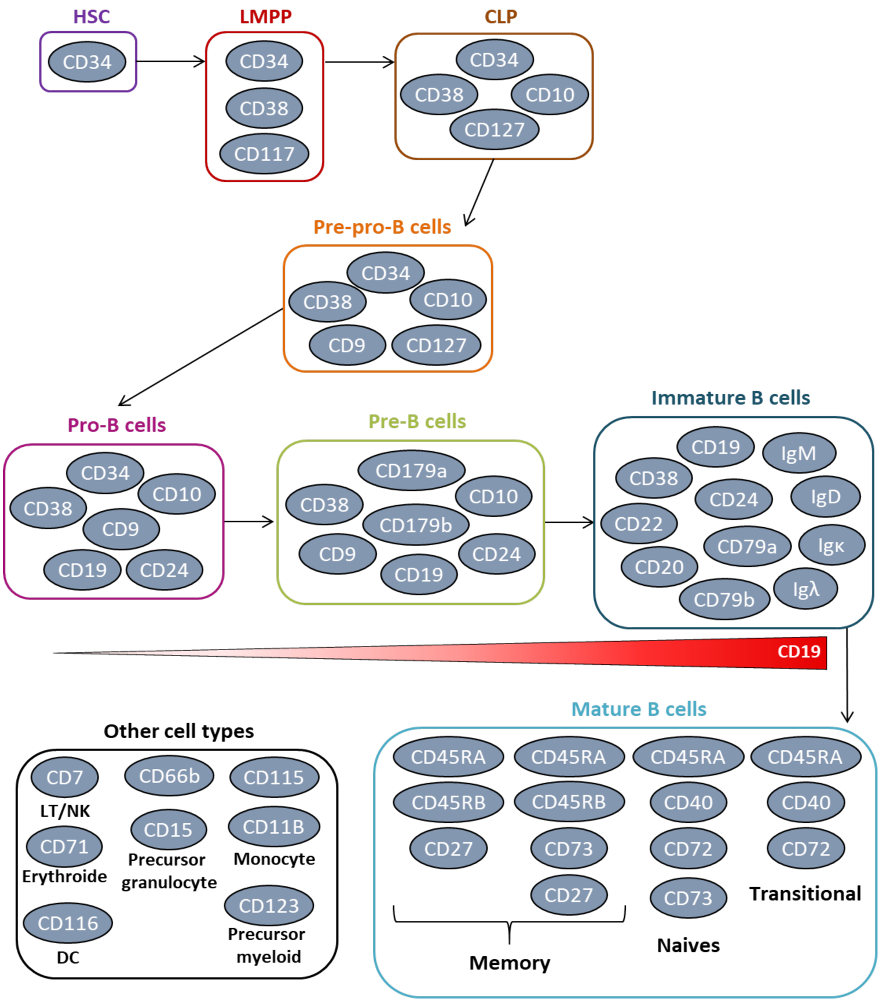

Figure S5:

A. Gating strategy for mass cytometry analyses of CD15 negative cells.

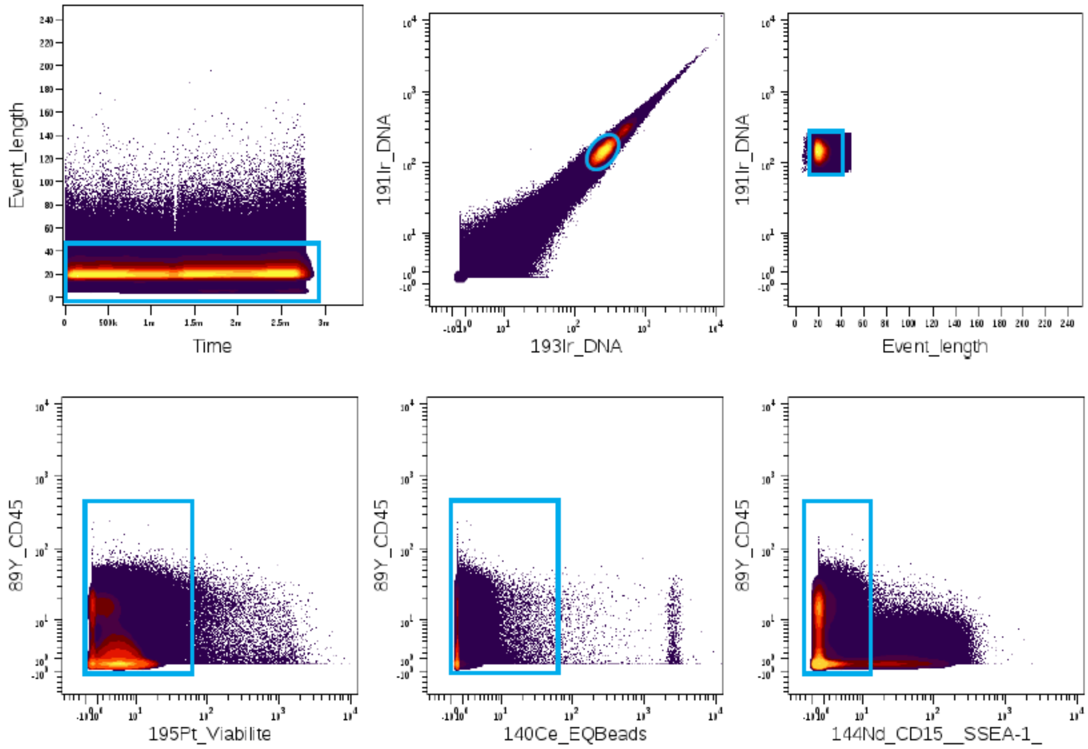

B. Gating strategy of early progenitors for mass cytometry analyses in CD15 negative cells.

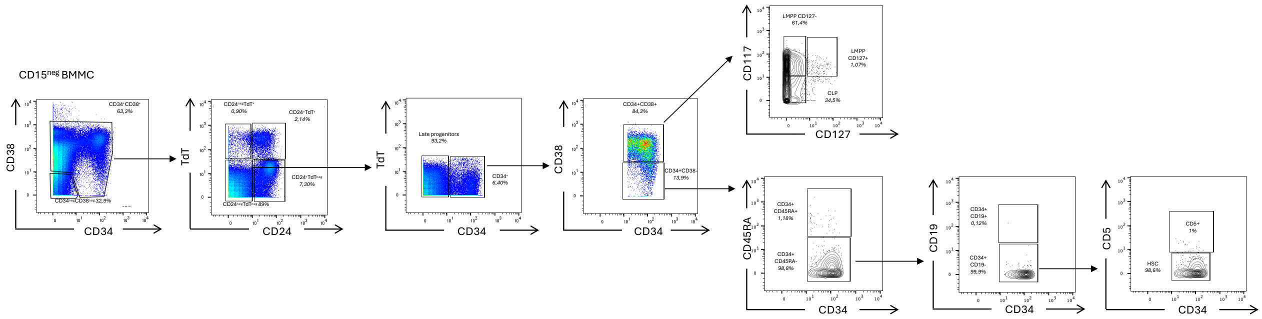

Figure S6: Key parameters associated with the PCA (Figure 2D)

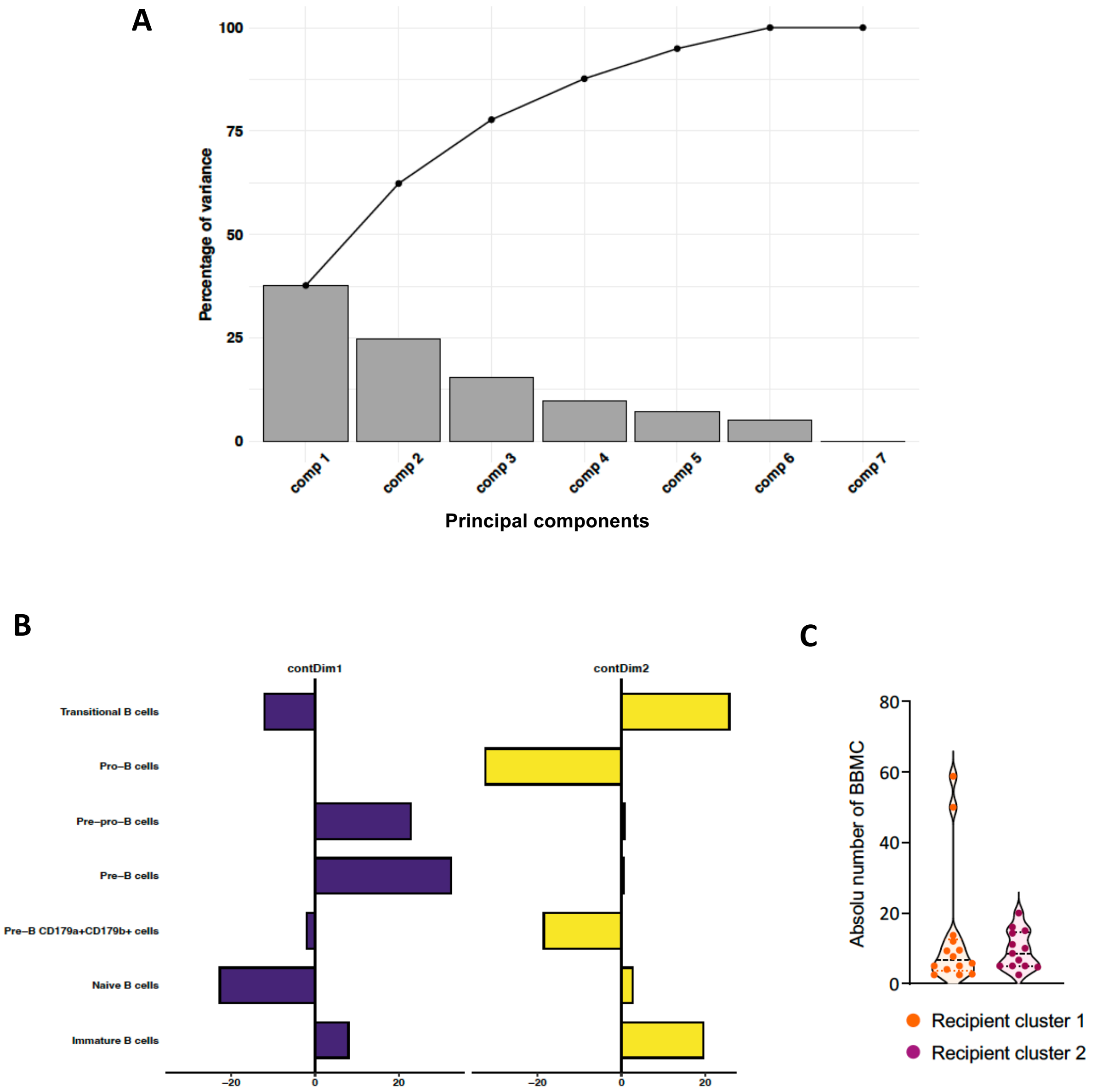

(**A**) Percentage of variance for each principal component. **(B)** Contribution of each B-cell subset to components 1 and 2. **(C)** Absolute bone marrow cell numbers in recipient of cluster 1 and recipient of cluster 2 (non-significant; Mann-Whitney test)

Figure S7: Clonal analyses of bone marrow B cells.

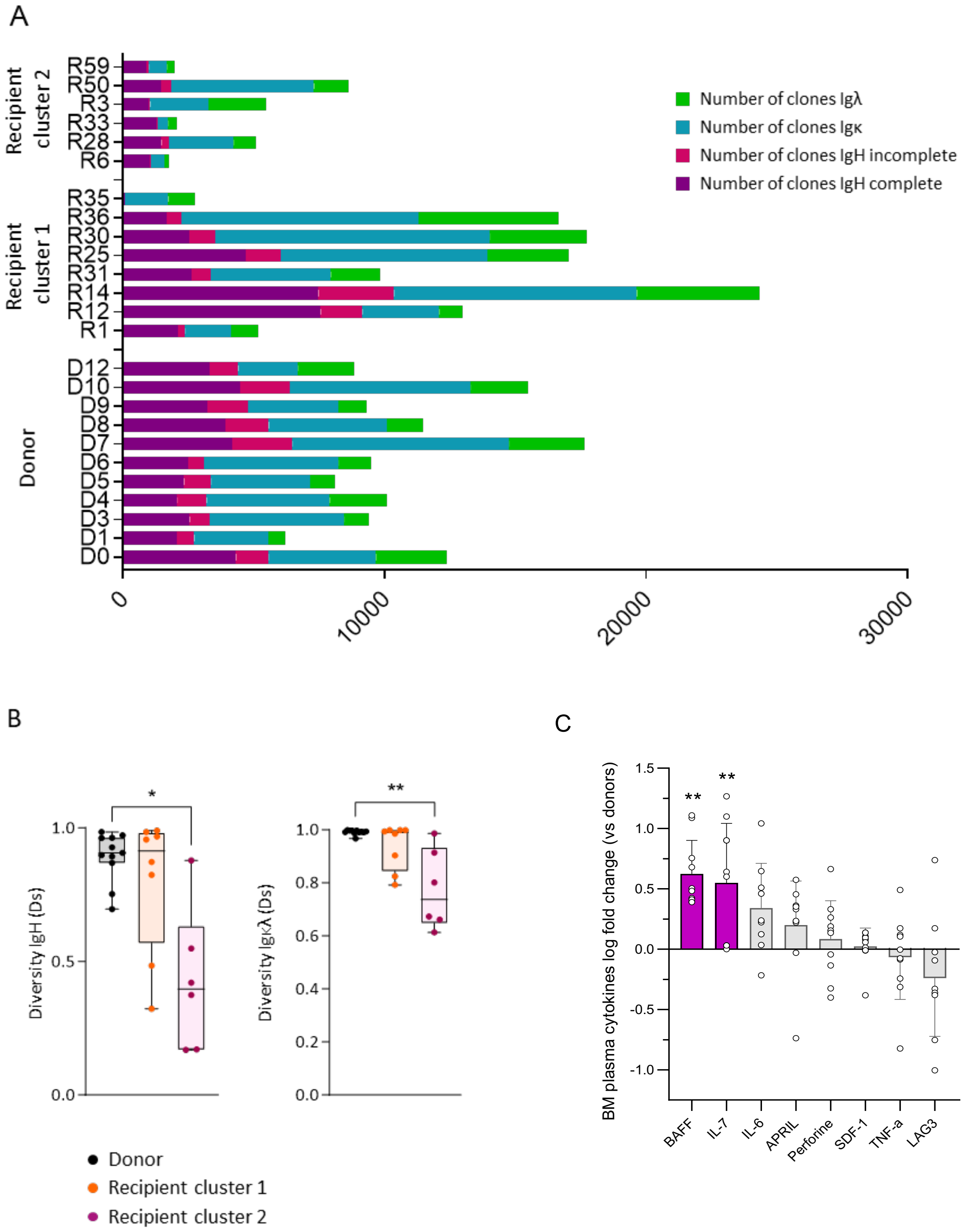

(A) Using next-generation sequencing (NGS) technology to quantify the number of clones of immunoglobulin light chains kappa (blue), immunoglobulin light chains lambda (green) and immunoglobulin heavy chains complete (purple) and incomplete (pink) per individual control (n=11), recipient cluster 1 (n=6) and recipient cluster 2 (n=8) patients. **(B)** Data showing the diversity (as assessed by the relative abundance of the clonotypes) of immunoglobulin heavy chains (IgH) and immunoglobulin light chains kappa/lambda (Igκλ) in controls, recipient cluster 1 and recipient cluster 2 patients. **(C)** Cytokines levels in bone marrow plasma studied by ELISA and Luminex from recipients (n=9) compared with controls (n=6). P-values correspond to Mann-Whitney-Wilcoxon test: *p <0.05, **p<0.01. Donor (D) refers to healthy control

Figure S8: Mass cytometry analyses of bone marrow T cells.

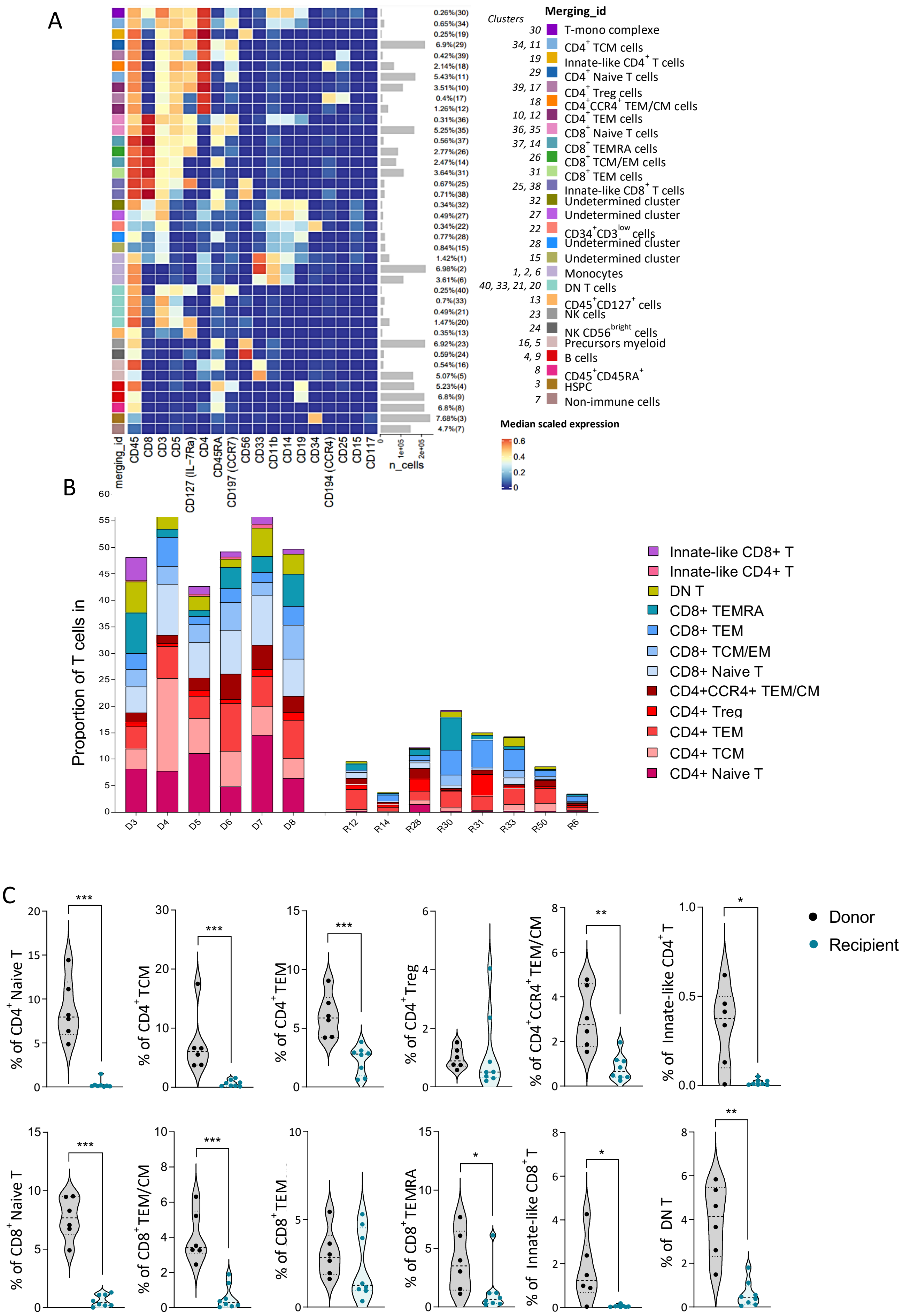

(A) Heatmap showing median expression intensities of each protein marker (columns), for each detected cluster (rows) using FlowSOM algorithm, with 41 indicated relevant metaclusters. **(B)** Data showing the proportion of T cells for all subsets per individual in controls (n=6) and recipients (n=8) patients. **(C)** Quantification of percentage of T cells for all subsets from bone marrow controls and recipients. Mann-Whitney tests: *p <0.05; **p<0.01; ***p<0.001. Donor (D) refers to healthy control

Figure S9: Mass cytometry analyses of bone marrow T cells in R1 and R2 and analyses of functional markers.

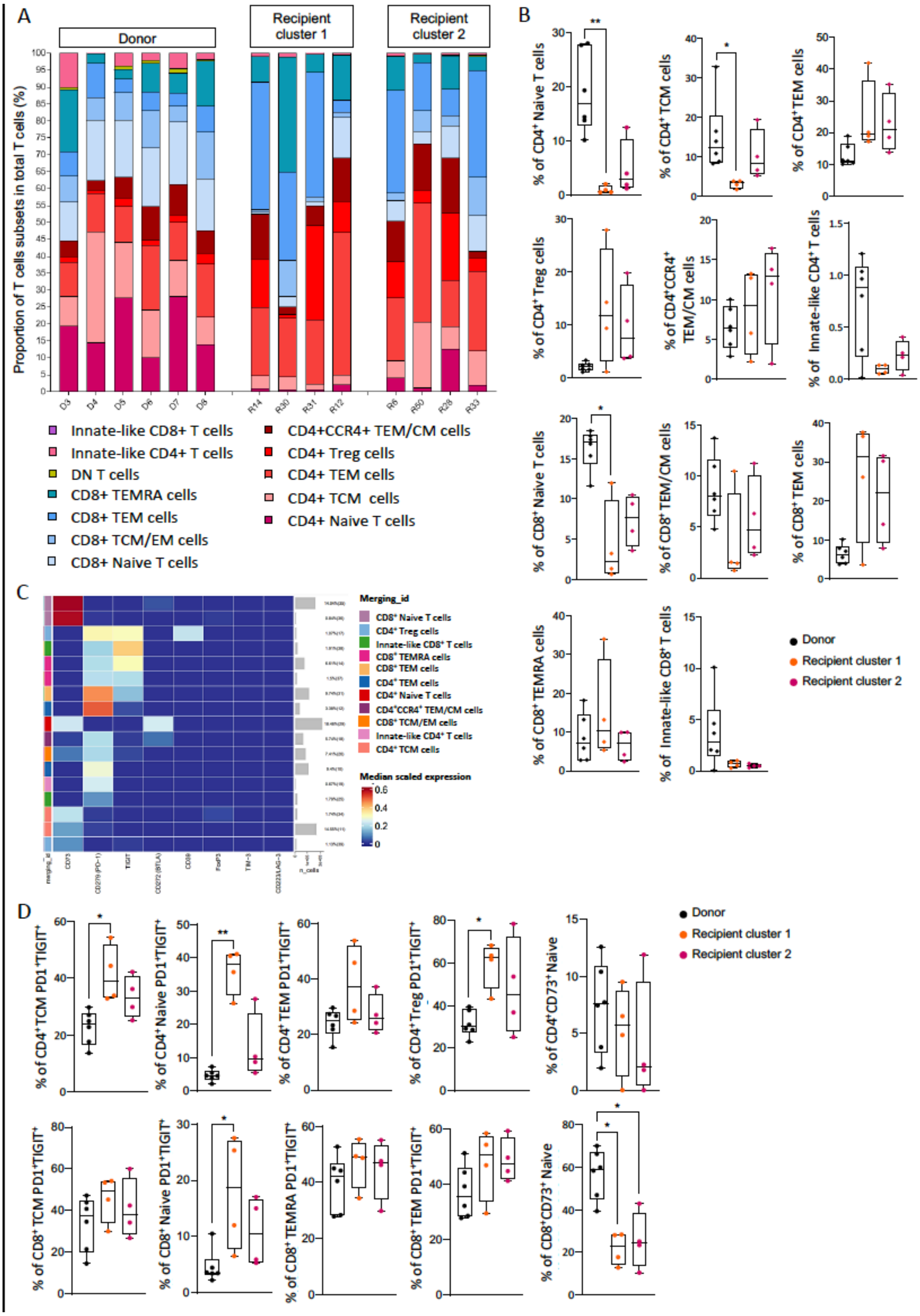

(A) Data showing the proportion of T cells for all subsets per individual in controls (n=6), recipients of cluster 1 (n=4) and recipients of cluster 2 (n=4) patients. **(B)** Quantification of percentage of T cells for all subsets from bone marrow controls, recipient of cluster 1 and recipient of cluster 2 patients. **(C)** Heatmap depicting scaled cell surface antigen expression in T-cell subsets. **(D)** Quantification of percentage of $PD1^{+}TIGIT^{+}$ and $CD73^{+}$ in T-cells subsets. P-values correspond to Mann-Whitney-Wilcoxon test: *p<0.05, **p<0.01. Donor (D) refers to healthy control

Figure S10: Representative B cell progenitors' associated TF expression projected on phenotypically defined cellular population (refer to TF not disclosed in Figure 5 & 6).

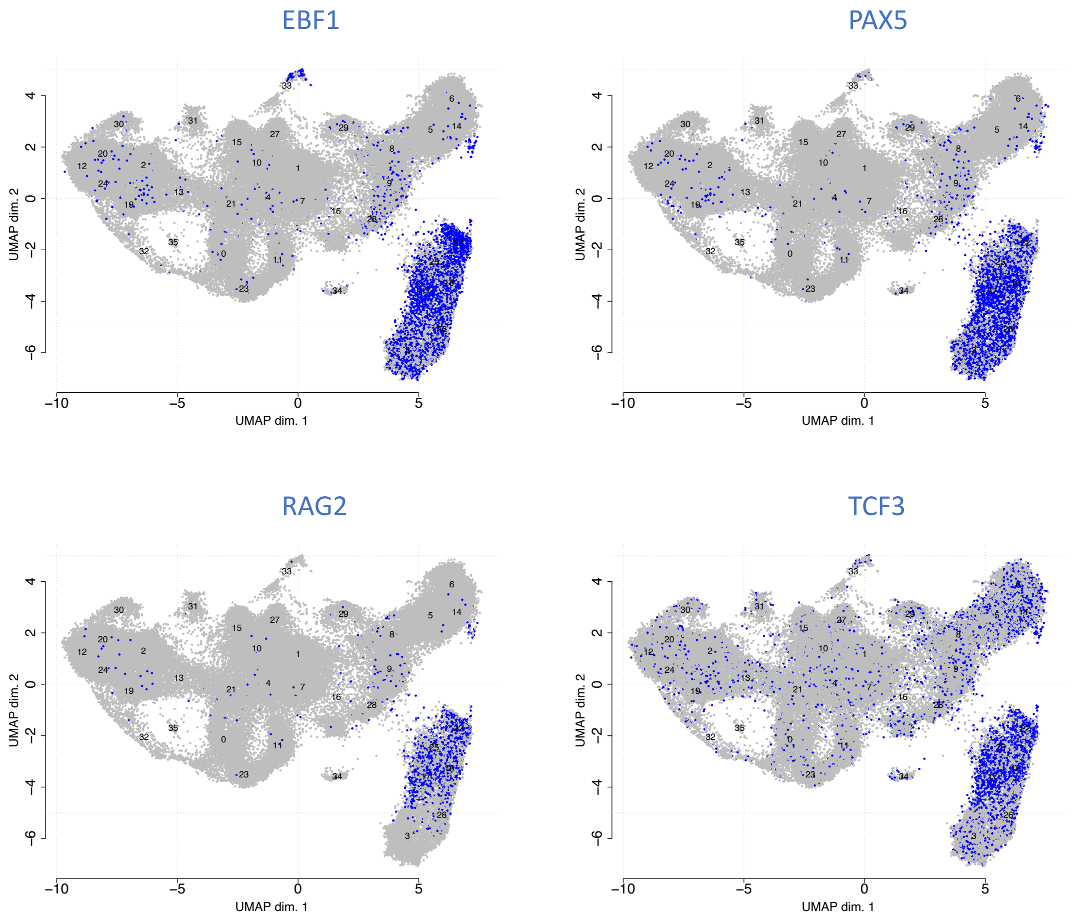

Figure S11: Bone marrow B-cells and myeloid progenitors.

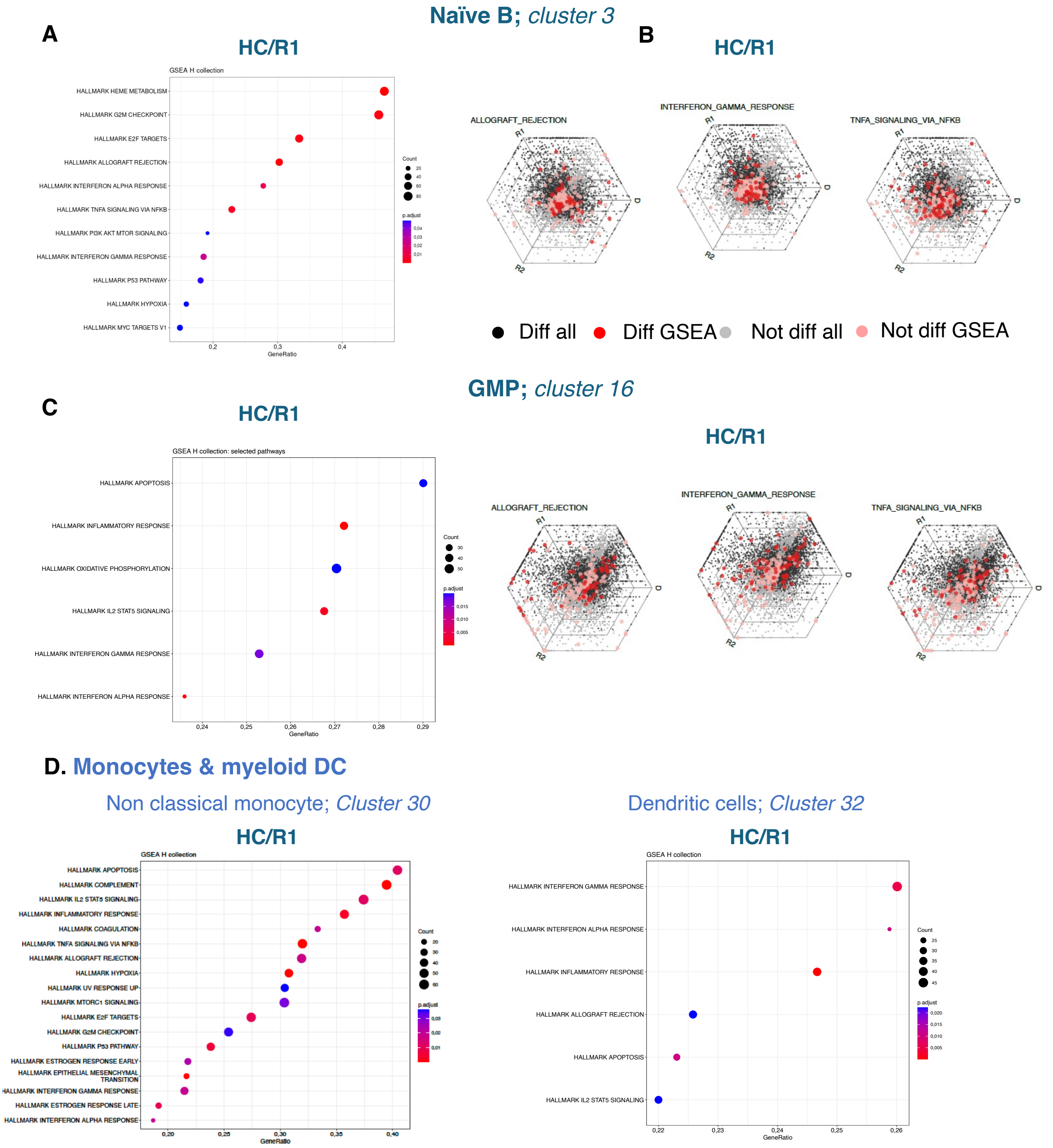

Legend to Supp Figure S11. **(A)** Functional enrichment analysis of annotated genes using hallmark collection. GSEA analysis based on logFC from limma-trend analysis on scran normalized data. Figures show significant (Benjamini-Hochberg, adjusted p-values <5%) functional enrichment in biological states or processes analysis in naïve B-cell **(A)**, granulo-monocytic progenitors (GMP) **(C)** and non-classical monocytes & myeloid DC2 **(D)**. **(B)** Hexagonal 'Triwise' plots displaying all arrayed genes for control (HC), recipient of group 1 (R1) with differentially expressed genes in HC/R1 (BJH adj. p-value < 0.05), depicted as a bigger red bullet for genes form GSEA Hallmarks sets (left: Allograft Rejection, middle: Interferon Gamma Response, right: TNFA Signaling via NFKB) and as a black smaller bullet for others. Non-differentially expressed genes are displayed with lower color saturation. Each grid line represents expression level on the log2 scale. HC/R1 hexagonal plots are displayed in naïve B-cells and GMP.

Figure S12: Bone marrow Erythroid and megakaryocytic progenitors.

CD34+ early ERP, Cluster 8

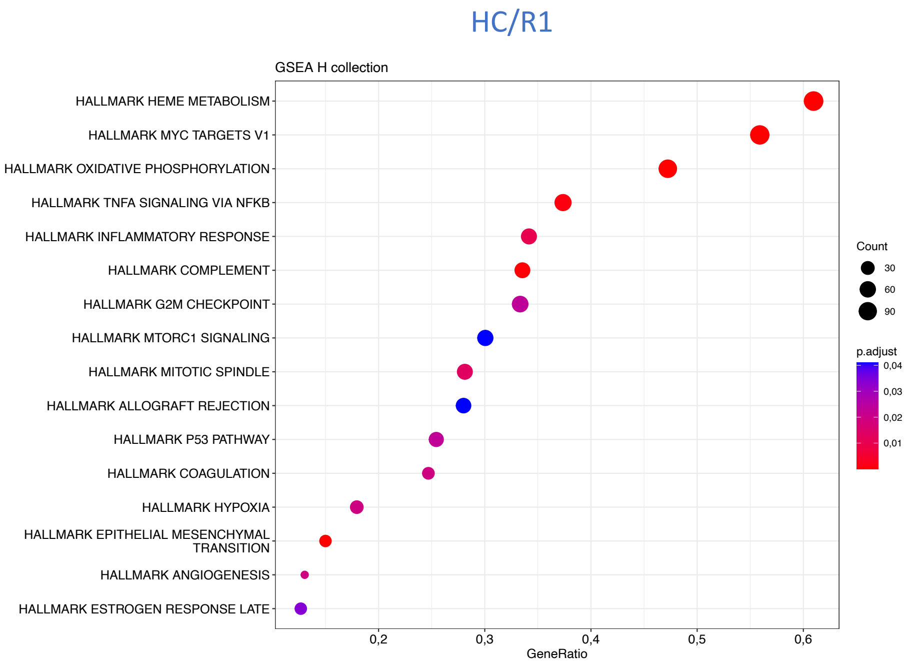

Erythroblast, cluster 14

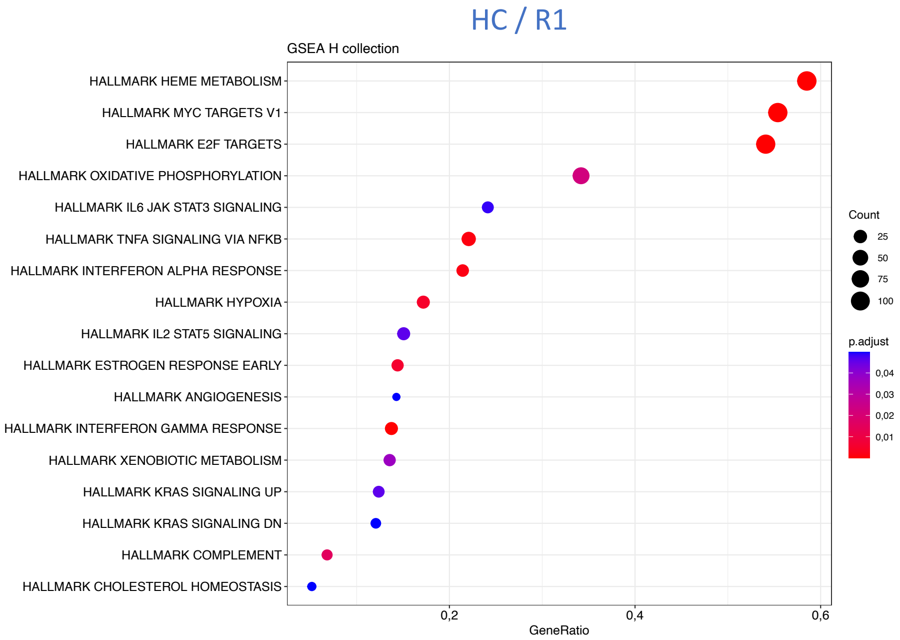

Legend to Supp Figure S12. Functional enrichment analysis of annotated genes using hallmark collection. GSEA analysis based on logFC from limma-trend analysis on scran normalized data. Figures show significant (Benjamini-Hochberg, adjusted p-values <0.05) functional enrichment in biological states or processes analysis in Erythroid and megakaryocytic progenitors (ERP) and in erythroblast between healthy control (HC) and recipient of group 1 (R1) patients.

Figure S13: Bone marrow NK-cells.

NKT, Cluster 11

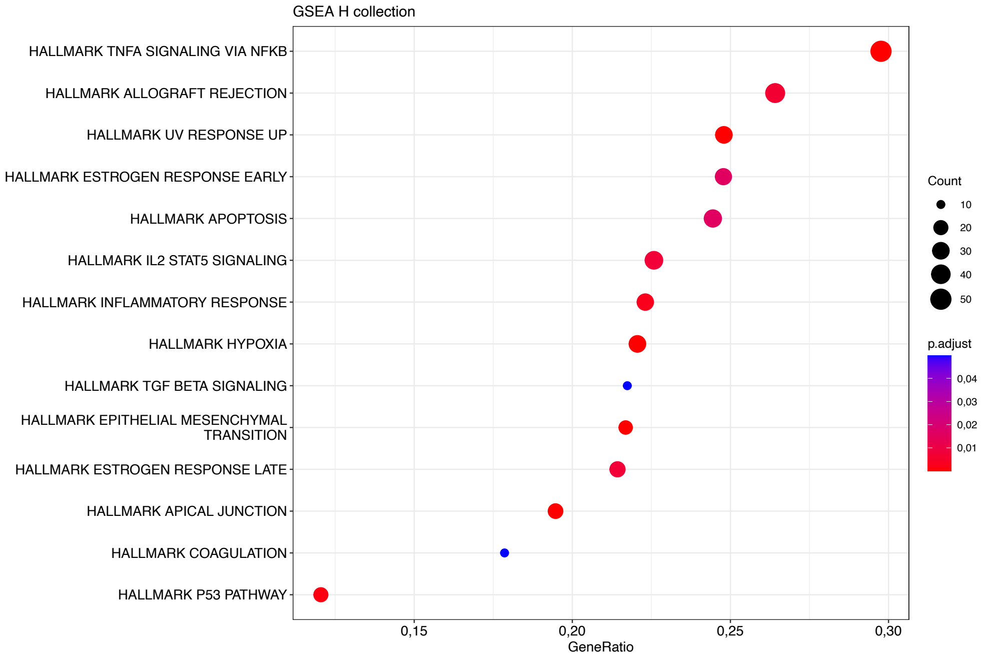

NK, Cluster 0

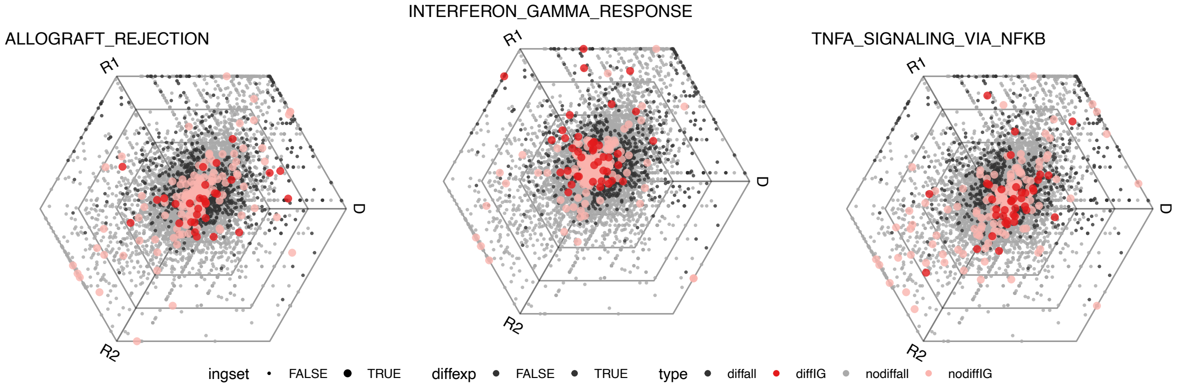

Legend to Supp Figure S13. Functional enrichment analysis of annotated genes using hallmark collection. GSEA analysis based on log-FC from limma-trend analysis on scran normalized data. Figures show significant (Benjamini-Hochberg, adjusted p-values <0.05) functional enrichment in biological states or processes analysis in NKT cells clusters between control and recipient of group 1 (R1) patients. Hexagonal 'Triwise' plots displaying all arrayed genes for control (HC), recipient of group 1 (R1) with differentially expressed genes in HC/R1 (BJH adj. p-value < 0.05), depicted as a bigger red bullet for genes form GSEA Hallmarks sets (left: Allograft Rejection, middle: Interferon Gamma Response, right: TNFA Signaling via NFKB) and as a black smaller bullet for others. Non-differentially expressed genes are displayed with lower color saturation. Each grid line represents expression level on the log2 scale. HC/R1 hexagonal plot is displayed in NK cells (cluster 0).

Figure S14: Exhaustion and Cytokines.

A

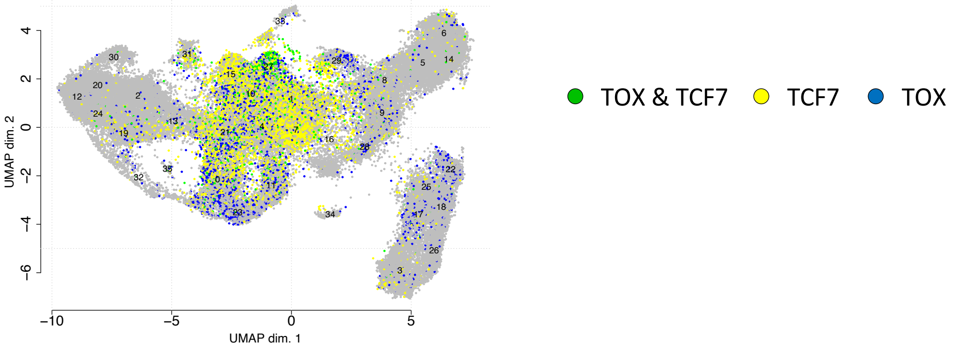

B

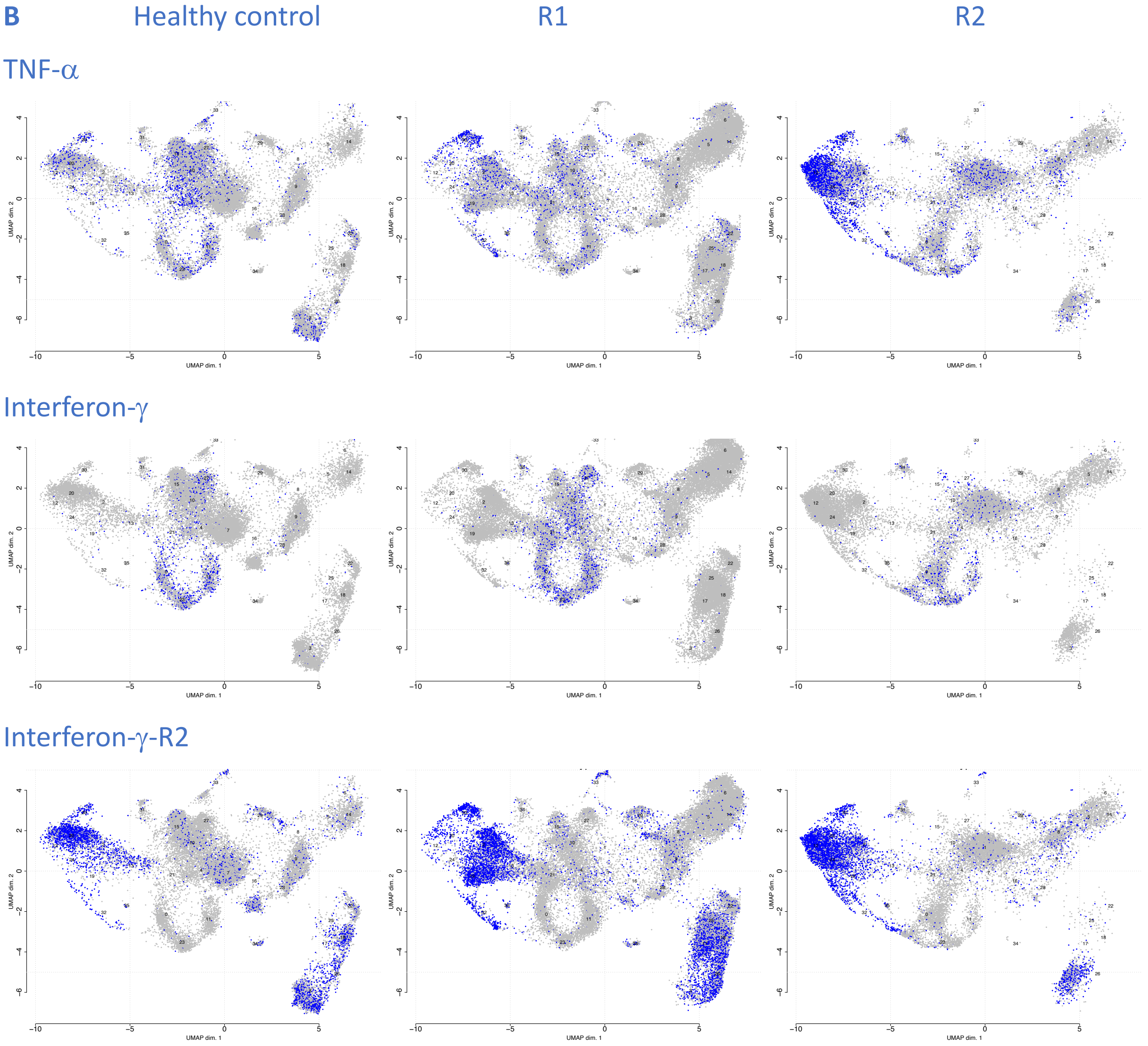

A. Expression of the TF TOX (blue), TC7 (yellow) or both (green) projected on the original UMAP.
B. Expression of selected mRNAs highlighted in blue on the UMAP from Fig.5A between healthy control, recipient of group 1 (R1) and recipient of group 2 (R2) patients.

Figure S15: GSEA hallmarks' dot plot summary comparing healthy controls to recipients R1.

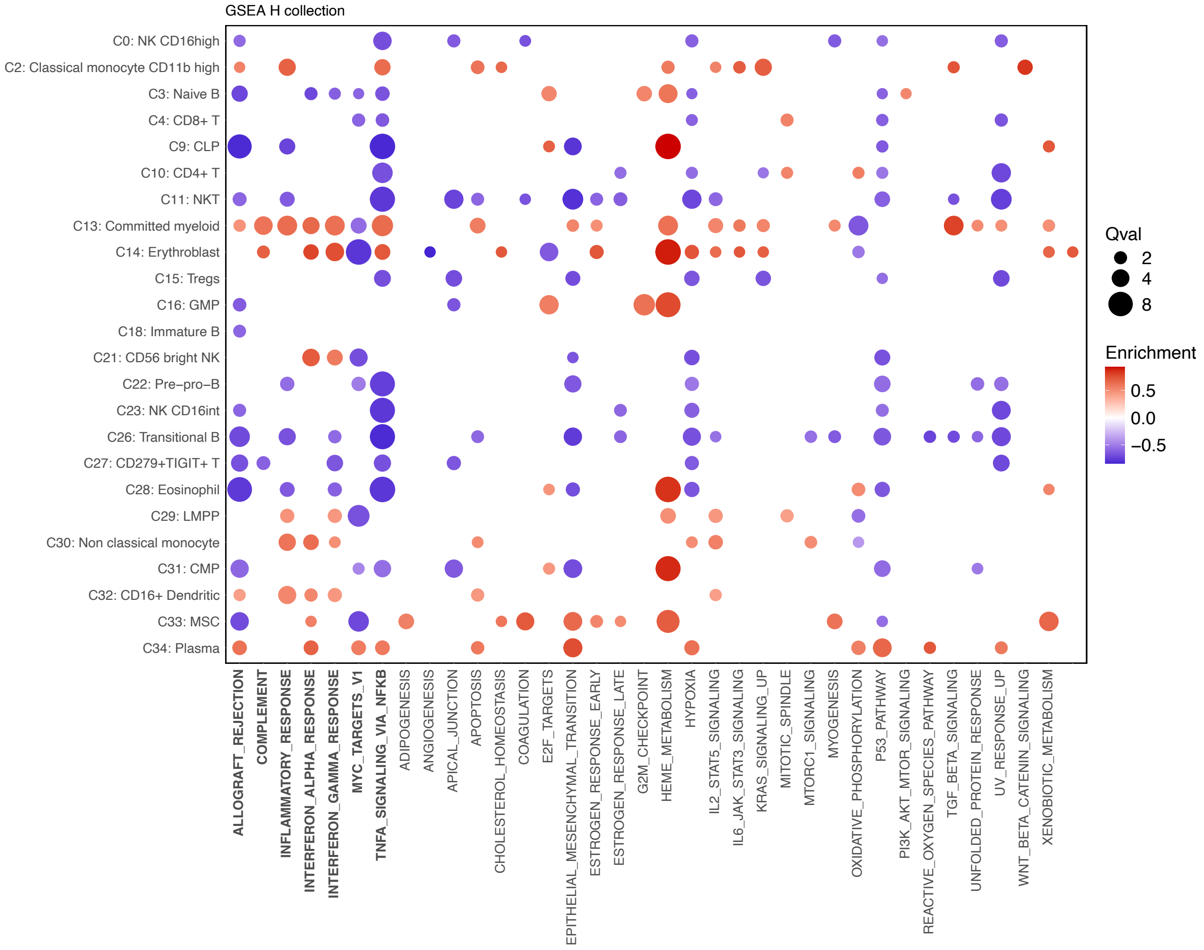

Figure S16: Overall summary of experimental results.

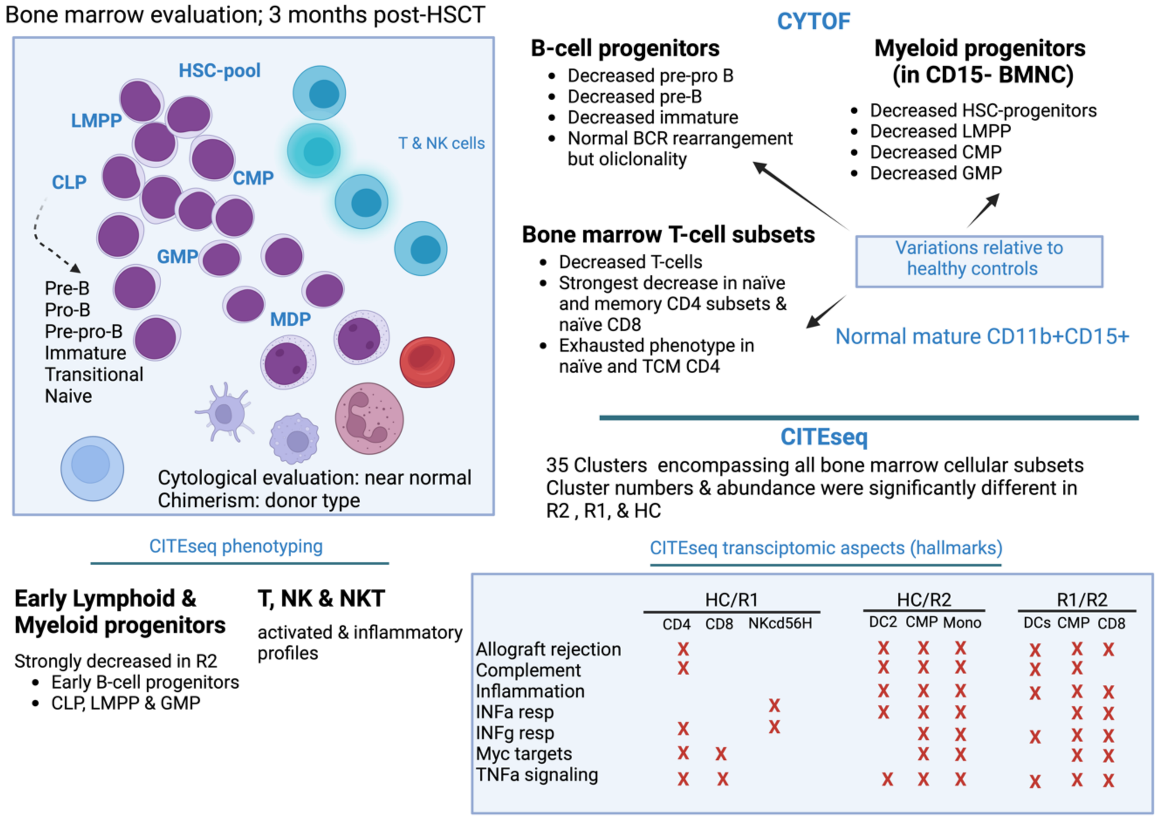

	HC/R1			HC/R2			R1/R2		
	CD4	CD8	NKcd56H	DC2	CMP	Mono	DCs	CMP	CD8
Allograft rejection	X			X	X	X	X	X	X
Complement	X			X	X	X	X	X	
Inflammation				X	X	X	X	X	X
INFa resp			X	X	X	X		X	X
INFg resp	X		X		X	X	X	X	X
Myc targets	X	X			X	X		X	X
TNFa signaling	X	X		X	X	X	X	X	X

Created with BioRender.com
